# Supplementary material for: Prefrontal signals precede striatal signals for biased credit assignment in motivational learning biases
Source: Nat Commun. 2024 Jan 2;15:19. doi: 10.1038/s41467-023-44632-x (PMC10762147; doi:10.1038/s41467-023-44632-x)
Supplement: Supplementary file 1 — Supplementary Information [file 41467_2023_44632_MOESM1_ESM.pdf]

Supplementary Information to "Prefrontal signals precede striatal signals for biased credit assignment in motivational learning biases"

Contents

Supplementary Note 1: Behavioral results with only the 29 participants included in EEG-fMRI analyses 3

Supplementary Note 2: Behavioral fMRI results with only the 29 participants included in EEG-fMRI analyses 5

Supplementary Note 3: EEG results with only the 29 participants included in EEG-fMRI analyses 8

Supplementary Note 4: EEG and fMRI correlates of past action with only the 29 participants included in EEG-fMRI analyses 10

Supplementary Note 5: Stay behavior as a function of EEG and fMRI with only the 29 participants included in EEG-fMRI analyses 12

Supplementary Note 6: Parameter recovery analyses for model M5 13

Supplementary Note 7: Simulations for asymmetric pathways and action priming model 15

Supplementary Note 8: Behavioral results for the perseveration model (M7), cue valence-based perseveration model (M8), and neutral outcomes reinterpretation model (M9) 17

Supplementary Note 9: Neural results based on prediction-errors from the cue valence-based perseveration model (M8) and neutral outcomes reinterpretation model (M9) 20

Supplementary Note 10: Illustration of biased and standard prediction error regressors for a representative example participant 22

Supplementary Note 11: Masks for fMRI analyses 24

Supplementary Note 12: EEG time-frequency results after ERPs were removed 26

Supplementary Note 13: ERPs as a function of action and outcome 28

Supplementary Note 14: Model-based EEG analyses in the time domain 31

Supplementary Note 15: Illustration of EEG-fMRI analysis fusion approaches 32

Supplementary Note 16: fMRI-informed EEG results in time-frequency space 34

Supplementary Note 17: fMRI-informed EEG results in the time domain 35

Supplementary Note 18: Go/NoGo differences over time in BOLD signal, choices, alpha, and beta power 37

Supplementary Note 19: Stay behavior as a function of BOLD and EEG TF power 39

Supplementary Table 1: Stay behavior as a function of action, salience, and valence 40

Supplementary Table 2: Model parameters and fit indices for models M1-M6.................................. 41
Supplementary Table 3: BOLD-GLM with parametric modulation by standard and biased prediction errors.. 42
Supplementary Table 4: BOLD-GLM with response-locked and outcome-locked categorical regressors... 43
Supplementary Table 5: Significant clusters in BOLD-GLM with parametric modulation by standard and biased prediction errors 44
Supplementary Table 6: Significant clusters in BOLD-GLM with response-locked and outcome-locked categorical regressors.. 46
Supplementary Table 7: Significant clusters in BOLD-GLM with EEG regressors............................ 48
Supplementary References 51

Supplementary Note 1: Behavioral results with only the 29 participants included in EEG-fMRI analyses

We repeated the behavioral analyses reported in the main text while excluding the seven participants that were also not included in the fMRI-inspired EEG analyses in the main text: (a) two participants due to fMRI co-registration failure, which were also not included in the fMRI-only analyses; (b) four further participants who exhibited excessive residual noise in their EEG data (> 33% rejected trials) and were thus also not included in the EEG-only analyses, and finally (c) one more participant who (together with four other participants already excluded) exhibited regression weights for every regressor about ten times larger than for other participants.

Participants in this subgroup learned the task, reflected in a significant main effect of required action on responses, $\chi^2(1) = 28.398$, $p < .001$, $b = 0.896$, *95%-CI* [0.643, 1.149], two-tailed, and exhibited motivational biases, reflected in a significant main effect of cue valence on responses, $\chi^2(1) = 19.308$, $p < .001$, $b = 0.439$, *95%-CI* [0.274, 0.604], two-tailed. The interaction between required action and cue valence was not significant, $\chi^2(1) = 0.111$, $p = .739$, $b = 0.025$, *95%-CI* [-0.142, 0.192], two-tailed (Supplementary Fig. 1A-B).

Participants in this subgroup also showed biased learning: They were more likely to repeat an action after a positive outcome (main effect of outcome valence: $\chi^2(1) = 40.920$, $p < .001$, $b = 0.553$, *95%-CI* [0.437, 0.669], two-tailed). After salient outcomes, they adjusted their responses more strongly after feedback on Go than on NoGo responses, in line with our model of biased learning and as reflected in a significant three-way interaction between action, salience, and valence, $\chi^2(1) = 16.862$, $p < .001$, $b = 0.266$, *95%-CI* [0.158, 0.374], two-tailed. When only analyzing trials with salient outcomes, outcome valence was more likely to affect response repetition following Go relative to NoGo responses, $\chi^2(1) = 13.266$, $p < .001$, $b = 0.324$, *95%-CI* [0.169, 0.479], two-tailed, with a stronger effect of outcome valence after Go responses, $\chi^2(1) = 49.003$, $p = .001$, $b = 1.342$, *95%-CI* [1.107, 1.577], two-tailed, than NoGo responses, $\chi^2(1) = 18.988$, $p < .001$, $b = 0.693$, *95%-CI* [0.440, 0.946], two-tailed (Supplementary Fig. 1C).

In this subgroup of participants, Bayesian model selection clearly favored the full asymmetric pathways models featuring response and learning biases (M5, model frequency: 81.81%, protected exceedance probability: 100%; Supplementary Fig. 1D-H). In sum, behavioral results were qualitatively identical when analyzing only this subgroup of only 29 participants.

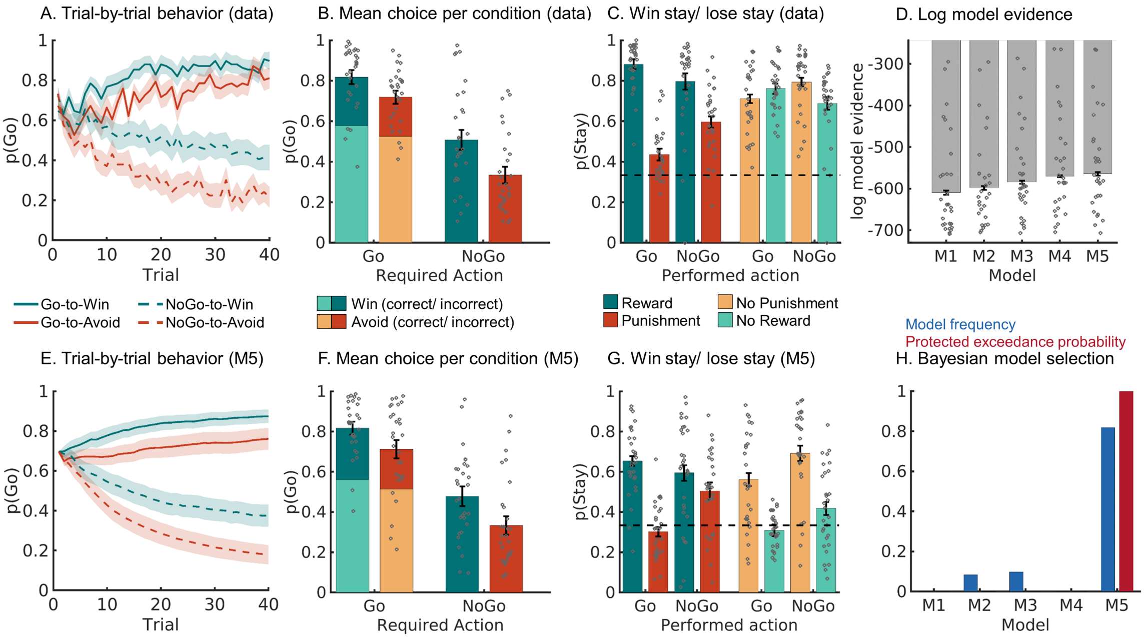

Supplementary Figure 1. Behavioral performance in the subgroup of 29 participants included in the fMRI-inspired EEG analyses. **A.** Trial-by-trial proportion of Go responses (error bands are ±SEM across participants, n=29) for Go cues (solid lines) and NoGo cues (dashed lines). The motivational bias was already present from very early trials onwards, as participants made more Go responses to Win than Avoid cues (i.e., green lines are above red lines). Additionally, participants clearly learn whether to make a Go response or not (proportion of Go responses increases for Go cues and decreases for NoGo cues). **B.** Mean (error bars are ±SEM across participants, n=29) proportion Go responses per cue condition (points are individual participants' means). **C.** Probability of repeating a response ("stay") on the next encounter of the same cue as a function of action and outcome (error bands are ±SEM across participants, n=29). Learning was reflected in higher probability of staying after positive outcomes than after negative outcomes (main effect of outcome valence). Biased learning was evident in learning from salient outcomes, where this valence effect was stronger after Go responses than NoGo responses. Dashed line indicates chance level choice ($p_{Stay} = 0.33$). **D.** Log-model evidence favors the asymmetric pathways model (M5 over simpler models (M1-M4; error bars are ±SEM across participants, n=29). **E-G.** Trial-by-trial proportion of Go responses, mean proportion Go responses, and probability of staying based on one-step-ahead predictions using parameters (hierarchical Bayesian inference) of the winning model (asymmetric pathways model, M5; error bars are ±SEM across simulated agents, n=29). **H.** Model frequency and protected exceedance probability indicate best fit for model M5 (asymmetric pathways model), in line with log model evidence.

Supplementary Note 2: Behavioral fMRI results with only the 29 participants included in EEG-fMRI analyses

We repeated the fMRI analyses reported in the main text while excluding the seven participants that were also not included in the fMRI-inspired EEG analyses in the main text: (a) two participants due to fMRI co-registration failure, which were also not included in the fMRI-only analyses; (b) four further participants who exhibited excessive residual noise in their EEG data (> 33% rejected trials) and were thus also not included in the EEG-only analyses, and finally (c) one more participant who (together with four other participants already excluded) exhibited regression weights for every regressor about ten times larger than for other participants.

We first repeated the model-free GLM just contrasting positive and negative outcomes. BOLD signal was higher for positive than negative outcomes in five clusters, namely in vmPFC, striatum, amygdala, and hippocampus (z_{max} = 5.65, p = 2.24e-25, 6110 voxels, MNI coordinates xyz = [6 30 -12]), left superior lateral occipital cortex (z_{max} = 4.40, p = .00144, 367 voxels, xyz = [-46 -68 46]), right occipital pole (z_{max} = 4.45, p = .00154, 363 voxels, xyz = [12 -92 -12]), posterior cingulate cortex (z_{max} = 4.36, p = .00181, 353 voxels, xyz = [-2 -48 28]), and left middle temporal gyrus (z_{max} = 4.63, p = .00548, 289 voxels, xyz = [-60 -10 -16]; Supplementary Fig. 2A). The clusters in left slOCC, PCC, and left MTG emerged anew compared to the original analysis comprising 34 participants. Also, compared to the original analysis, clusters in left orbitofrontal cortex and left superior frontal gyrus were merged with the cluster in vmPFC. In sum, all clusters from the original analysis were found back, plus some additional clusters.

There was also one cluster in right orbitofrontal cortex (z_{max} = 4.37, p = .0209, 217 voxels, xyz = [30 62 -2]) in which BOLD signal was higher for negative than positive outcomes. Compared to the original analysis comprising 34 participants, clusters in precuneus and right superior frontal gyrus were not significant.

In the model-based GLM featuring regressors for standard PEs and the difference term towards biased PEs, BOLD signal correlated with standard PEs in ten clusters, namely in vmPFC, striatum, bilateral amygdala and hippocampus (z_{max} = 6.04, p = .4.78e-44, 8848 voxels, xyz = [12 14 -6]), left superior frontal gyrus (z_{max} = 5.58, p = 3.5e-10, 1043 voxels, xyz = [-18 34 52]), left occipital pole and lingual gyrus (z_{max} = 6.23, p = 7.18e-10, 998 voxels, xyz = [10 -92 -10]), posterior cingulate cortex (z_{max} = 5.12, p = 8.57e-10, 987 voxels, xyz = [4 -36 48]), left inferior temporal gyrus (z_{max} = 5.03, p = 7.07e-09, 859 voxels, xyz = [-52 -46 -10]), right anterior middle temporal gyrus (z_{max} = 5.32, p = .000292, 314 voxels, xyz = [62 -4 -16]), right cerebellum (z_{max} = 5.32, p = .002228, 231 voxels, xyz = [44 -72 -40]), left superior lateral occipital cortex (z_{max} = 4.69, p = .00322, 218 voxels, xyz = [-46 -74 -38]), right caudate (z_{max} = 4.33, p = .00538, 199 voxels, xyz = [20 12 22]), and right middle temporal gyrus (z_{max} = 4.09, p = .0129, 189 voxels, xyz = [54 -38 -12] ; Supplementary Fig. 2B). The clusters in left superior lateral occipital cortex, right caudate, and right posterior middle temporal gyrus emerged anew by splitting from larger clusters visible in the original analysis based on 34 participants. Vice versa, the cluster in left middle temporal gyrus reported for the original analysis was merged with a bigger cluster in the analysis of only 29 participants. The clusters in postcentral gyrus and ACC observed in the original analysis based on 34 participants were not significant anymore; however, they were still visible at a level of z > 3.1 uncorrected.

BOLD signal correlated significantly negatively with standard PEs in a single cluster in right superior frontal gyrus (z_{max} = 5.04, p = .00771, 186 voxels, xyz = [6 26 64]), similar to the respective cluster reported in the original analysis. In contrast, the clusters in right occipital pole, intracalcarine cortex, and left inferior lateral occipital cortex were not significant any more, though visible at a level of z > 3.1 uncorrected.

BOLD signal in six clusters correlated significantly positively with the difference term towards biased PEs, namely in large parts of cortex and subcortex including striatum (z_{max} = 6-54, p = 0, 29428 voxels, xyz = [34 -84 20]), dorsomedial prefrontal cortex (z_{max} = 5.94, p = 2.69e-40, 7001 voxels, xyz = [6 22 34]), right insula (z_{max} = 5.76, p = 7.84e-27, 3847 voxels, xyz = [34 20 -8]), thalamus and brainstem (z_{max} = 5.10, p = 4.06e-18, 2169 voxels, xyz = [4 -30 0]), left caudate (z_{max} = 4.71, p = .000188, 305 voxels, xyz = [-12 8 6]) and another cluster in brainstem (z_{max} = 4.05, p = .0151, 160 voxels, xyz = [4 -30 -30]). Clusters in dmPFC, right insula, and left caudate split from larger clusters reported in the original analysis. Vice versa, the cluster in left insula reported in the original analysis merged with the largest cluster. The clusters in right middle temporal gyrus and right insula were missing in the analysis of only 29 participants, but visible at a level of $z > 3.1$ uncorrected.

BOLD signal in three clusters correlated significantly negatively with the difference term towards biased PEs, namely in vmPFC (z_{max} = 4.23, p = .0051, 185 voxels, xyz = [-12 48 -6]), left hippocampus (z_{max} = 4.58, p = .00857, 168 voxels, xyz = [-26 -14 -22]), and left medial temporal gyrus (z_{max} = 4.30, p = .0172, 146 voxels, xyz = [-62 -4 -16]). Compared to the original analysis, the cluster in vmPFC emerged anew.

When computing the conjunction between both (positive) contrasts, BOLD signal encoded both the standard and the difference in four clusters, namely in vmPFC, bilateral striatum, bilateral ITG, and V1 (Supplementary Fig. 2C). Clusters in ACC, left motor cortex, and PCC were not significant any more (because they were $z > 3.1$, but not significant after cluster correction in the standard PE contrast). However, new (though rather small) clusters of biased PE encoding emerged in right insula, left amygdala, and left OFC. In sum, results when analyzing only this subgroup of only 29 participants were largely similar to results based on the full sample; however, clusters of biased PE encoding in left motor cortex, ACC, and PCC were small and thus did not survive cluster correction in this subgroup.

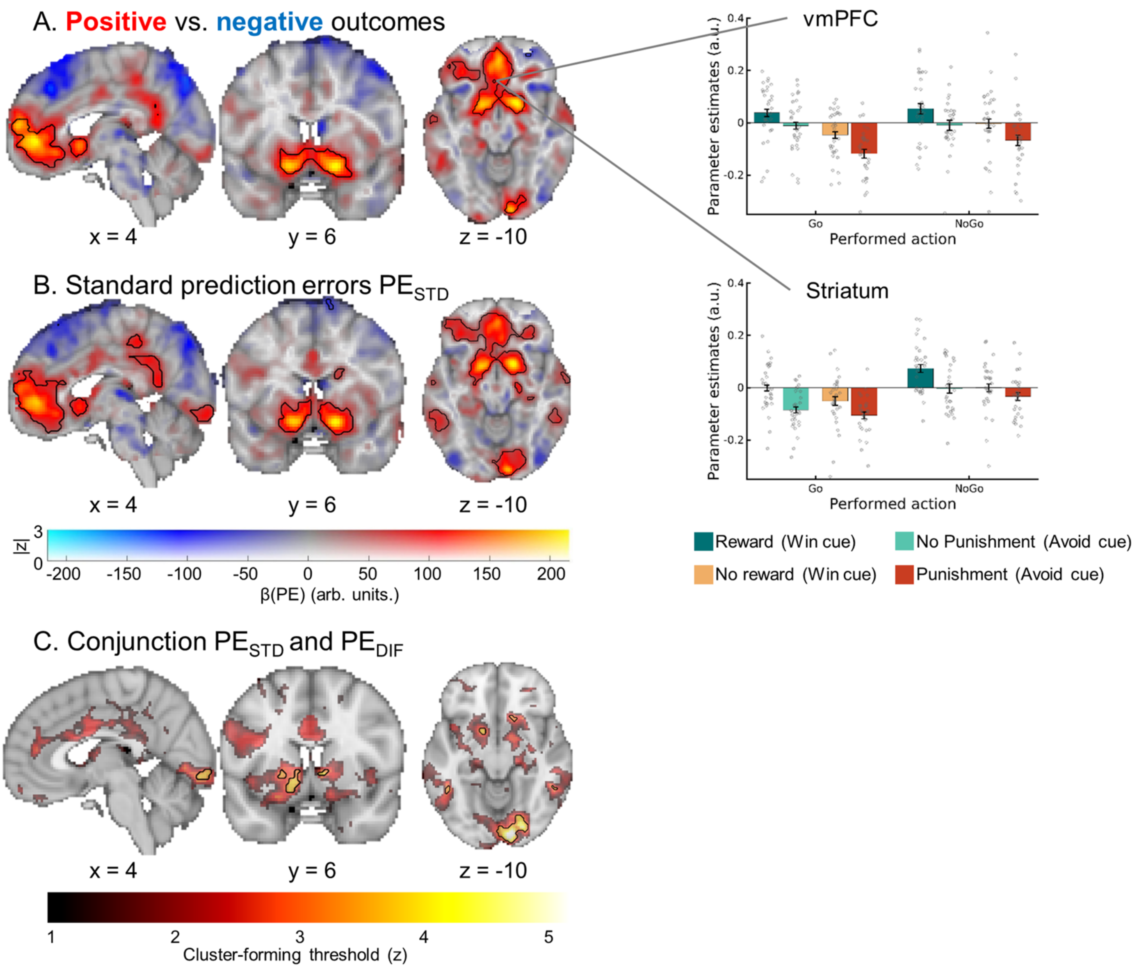

Supplementary Figure 2. BOLD signal reflecting outcome processing in the subgroup of 29 participants included in the fMRI-inspired EEG analyses. **A.** BOLD signal was higher for positive outcomes (rewards, no punishments) compared with negative outcomes (no rewards, punishments) in a range of regions including bilateral ventral striatum and vmPFC. BOLD effects displayed using a dual-coding data visualization approach with color indicating the parameter estimates and opacity the associated z-statistics. Significant clusters are surrounded by black edges. Bar plots show parameter estimates per action x outcome condition (error bars are ±SEM across participants, n=29) **B.** When using the trial-by-trial PEs participants experienced as model-based regressors in our GLM, positive PE correlations occurred in several regions including importantly the ventral striatum, vmPFC, dACC, and PCC. **C.** Left panel: Regions encoding both the standard PE term and the difference term to biased PEs (conjunction) at different cluster-forming thresholds (color). Clusters significant at a threshold of $z > 3.1$ are surrounded by black edges. In bilateral striatum, pgACC, bilateral ITG, and primary visual cortex, BOLD was significantly better explained by biased learning than by standard learning. Clusters in dACC, left motor cortex, and PCC were not significant any more.

Supplementary Note 3: EEG results with only the 29 participants included in EEG-fMRI analyses

We repeated the EEG analyses reported in the main text while excluding the seven participants that were also not included in the fMRI-inspired EEG analyses in the main text: (a) two participants due to fMRI co-registration failure, which were also not included in the fMRI-only analyses; (b) four further participants who exhibited excessive residual noise in their EEG data (> 33% rejected trials) and were thus also not included in the EEG-only analyses, and finally (c) one more participant who (together with four other participants already excluded) exhibited regression weights for every regressor about ten times larger than for other participants.

In participants in this subgroup, both midfrontal theta and beta power reflected outcome valence: Theta power was higher for negative than positive outcomes (driven by a cluster around 225–500 ms, p = .002, two-tailed; Supplementary Fig. 3A, B), while beta power was higher for positive than negative outcomes (driven by a cluster around 325–1000 ms, p = .002, two-tailed; Supplementary Fig. 3A, C). When using PE terms as regressor for midfrontal EEG power while controlling for PE valence, delta power did not encode PE_{STD} positively, though not significant (p = .056, two-tailed), and also the positive encoding of PE_{DIF} was non-significant (p = .053, two-tailed; Supplementary Fig. 3D-F). The positive correlation of beta power with PE_{STD} was not significant anymore (p = .059, two-tailed), while the negative correlation with PE_{DIF} remained (p = .001, two-tailed, 450–950 ms). When adding PE_{STD} and PE_{DIF} together to achieve PE_{BIAS}, theta/delta power indeed significantly encoded PE_{BIAS}, first positively (p = .032, two-tailed, 224–475 ms, two-tailed) and then negatively (p = .019, two-tailed, 600 – 1,000 ms, two-tailed; around 8 Hz and thus rather in the alpha band). Also, beta power was significantly negatively correlated with PE_{BIAS} (p = .008, two-tailed, 450 – 975 ms, two-tailed).

In sum, all findings reported in the main text also held when analyzing only this subgroup of only 29 participants. In addition, also late beta power and theta/alpha power appeared to negatively encode the PE_{BIAS} term.

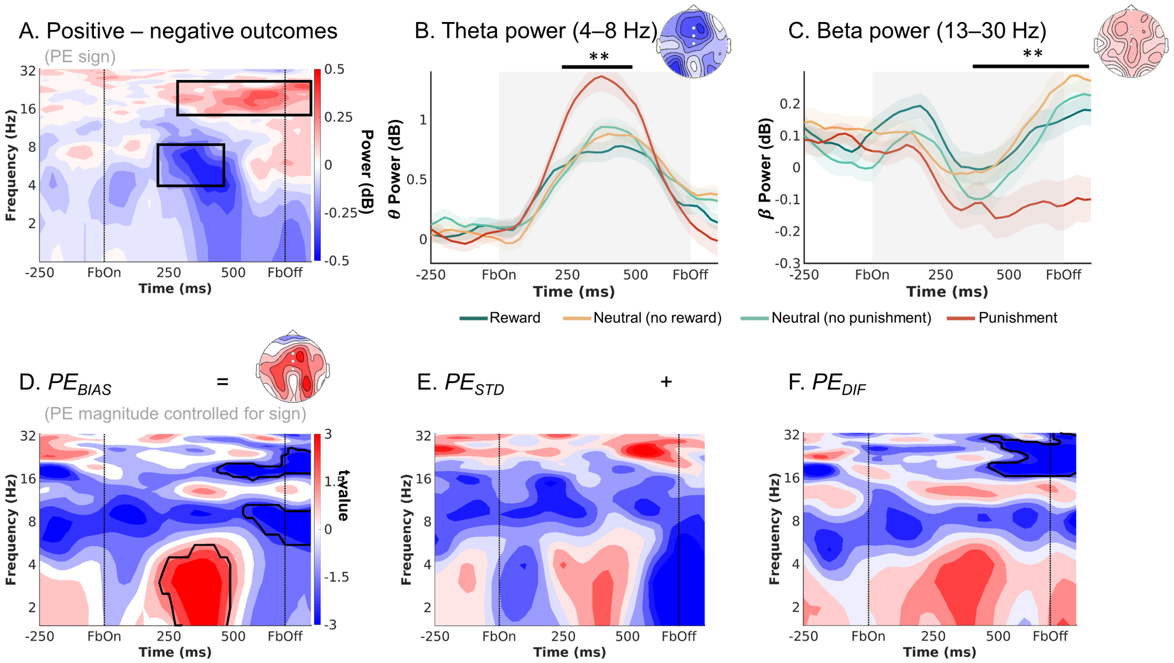

Supplementary Figure 3. EEG time-frequency power midfrontal electrodes (Fz/ FCz/ Cz) reflecting outcomes processing in the subgroup of 29 participants included in the fMRI-inspired EEG analyses. **A.** Time-frequency plot (logarithmic y-axis) displaying high theta (4–8 Hz) power for negative outcomes and higher beta power (16–32 Hz) for positive outcomes. **B.** Theta power transiently increases for any outcome, but more so for negative outcomes (especially punishments) around 225–475 ms (p = .002, two-tailed) after feedback onset (error bands are ±SEM across participants, n=29). **C.** Beta was higher for positive than negative outcomes (especially punishments) over a long time period around 300–1,250 ms (p = .002, two-tailed) after feedback onset (error bands are ±SEM across participants, n=29). **D-F.** Correlations between midfrontal EEG power and trial-by-trial PEs. Solid black lines indicate clusters above threshold. Biased PEs were significantly positively correlated with midfrontal theta power, but also negatively correlated with later alpha and beta power (**D**). The correlations of theta with the standard PEs (**E**) and the difference term to biased PEs (**F**) were also positive, though not significant. Beta power only encoded the difference term to biased PEs (**F**). ** $p < 0.01$, cluster-based permutation test.

Supplementary Note 4: EEG and fMRI correlates of past action with only the 29 participants included in EEG-fMRI analyses

We repeated the behavioral analyses reported in the main text while excluding the seven participants that were also not included in the fMRI-inspired EEG analyses in the main text: (a) two participants due to fMRI co-registration failure, which were also not included in the fMRI-only analyses; (b) four further participants who exhibited excessive residual noise in their EEG data (> 33% rejected trials) and were thus also not included in the EEG-only analyses, and finally (c) one more participant who (together with four other participants already excluded) exhibited regression weights for every regressor about ten times larger than for other participants.

Regarding fMRI correlates of the past action, similar to the original analysis comprising 34 participants, there were no clusters with higher BOLD after Go than NoGo actions at the time of outcomes, but vice versa, large parts of cortex and subcortex showed higher BOLD after NoGo than Go actions, highly similar to the original analysis (z_{max} = 7.65, p = 0, 124629 voxels, xyz = [-58 18 22]; Supplementary Fig. 4D).

Furthermore, there were four clusters with higher BOLD for Go than NoGo actions at the time of the response, namely one large cluster across lateral prefrontal cortex, anterior cingulate cortex, striatum, thalamus, angular gyrus, cerebellum, left operculum and motor cortex, intracalcarine cortex, and occipital pole (z_{max} = 7.45, p = 0, 61057 voxels, xyz = [32 -4 -4]), one in right middle temporal gyrus (z_{max} = 4.90, p = 8.66e-05, 493 voxels, xyz = [66 -32 -12]), one in left inferior temporal gyrus (z_{max} = 4.43, p = .00294, 293 voxels, xyz = [-60 -44 -18]), and one in precuneous (z_{max} = 2.39, p = .0041, 276 voxels, xyz = [-8 -70 38]; Supplementary Fig. 4C). All these regions were also found in the original analysis comprising 34 participants. Vice versa, BOLD signal was higher NoGo than Go actions at the time of the response in two clusters in vmPFC and subcallosal cortex (z_{max} = 4.23, p = .00864, 239 voxels, xyz = [-2 18 -6]) and right anterior temporal gyrus/ temporal pole (z_{max} = .4.14, p = .0193, 201 voxels, xyz = [48 -6 -8]), identical to the original analysis comprising 34 participants.

Finally, there was higher BOLD signal for left hand compared to right hand responses at the time of response in two clusters in right precentral and postcentral gyrus, superior parietal lobule, and operculum (z_{max} = 6.66, p = 0, 11597 voxels, xyz = [46 -24 64]) and left cerebellum (z_{max} = 6.76, p = 1.05e-18, 2672 voxels, xyz = [-18 -54 -16]; Supplementary Fig. 4C), identical to the original analysis comprising 34 participants. Vice versa, there was higher BOLD signal for right hand than left hand responses at the time of responses in five clusters in left precentral and postcentral gyrus, superior parietal lobule, operculum, and thalamus (z_{max} = 6.4, p = 0, 12372 voxels, xyz = [-36 -20 66]), right cerebellum (z_{max} = 7.17, p = 3.41e-21, 3206 voxels, xyz = [20 -54 -20]), right superior lateral occipital cortex (z_{max} = 4.84, p = 2.28e-09, 988 voxels, xyz = [48 -86 -4]), right angular gyrus (z_{max} = 4.11, p = 7.68e-05, 396 voxels, xyz = [66 -50 28]), and left superior lateral occipital cortex (z_{max} = 5.03, p = .019, 164 voxels, xyz = [-18 -82 48]). The clusters in right occipital pole/ intracalcarine cortex and in right posterior cerebellum observed in the original analysis comprising 34 participants were not observed in this analysis. In sum, all major findings also held when analyzing only this subgroup of only 29 participants.

Regarding EEG time-frequency correlates of the past action, when testing for differences in broadband after outcome onset, there was no significant difference after Go and NoGo responses, p = .283. When restricting analyses to the low alpha range, the permutation test was marginally significant, p = .056, driven by a cluster around 0–100 ms around 7–10 Hz; Supplementary Fig. 4A, B). When repeating the permutation test for the broadband signal including the last second before outcome onset, there was a significant difference after Go and NoGo responses, driven by clusters in the beta band. p = 0.002, -1000 – -275 ms, 13–32 Hz, and in the theta/ low alpha band, p = 0.020, -1000 – -525 ms, 4–10 Hz.

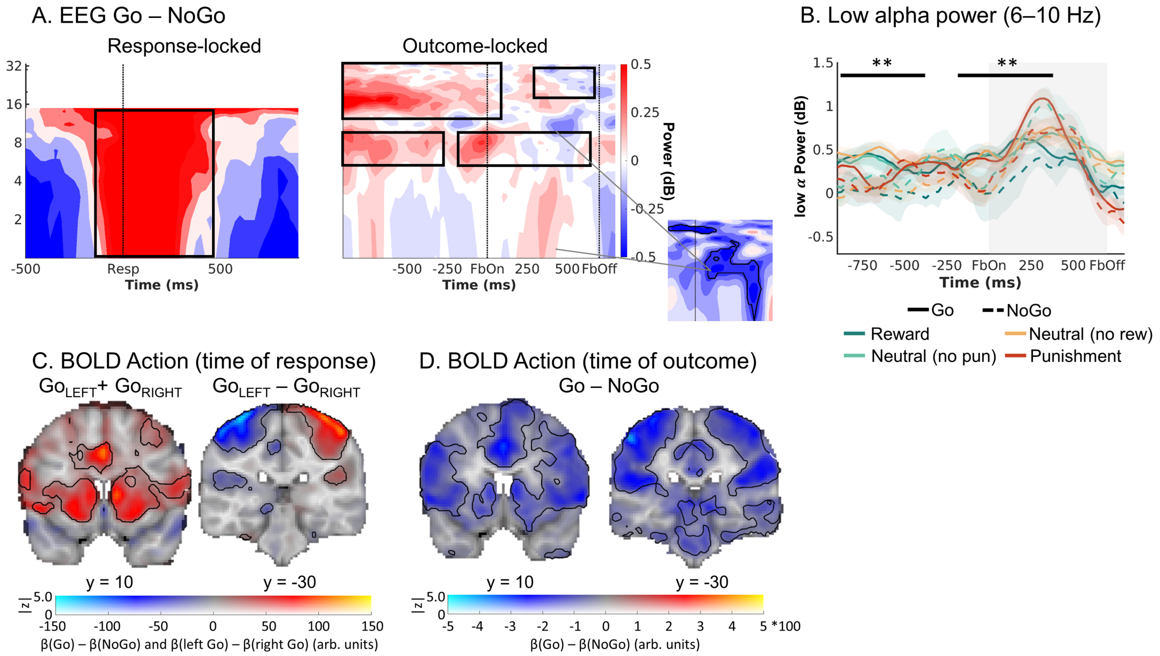

Supplementary Figure 4. Exploratory follow-up analyses on dACC BOLD signal and midfrontal low-alpha power in the subgroup of 29 participants included in the fMRI-inspired EEG analyses. **A.** Midfrontal time-frequency response-locked (left panel) and outcome-locked (right panel). Before and shortly after outcome onset, power in the lower alpha band was higher on trials with Go actions than on trials with NoGo actions. The shape of this difference resembles the shape of dACC BOLD-EEG TF correlations (small plot; note that this plot depicts BOLD-EEG correlations, which were negative). Note that differences between Go and NoGo trials occurred already before outcome onset in the alpha and beta range, reminiscent of delay activity; but were not fully sustained since the actual response. **B.** Midfrontal power in the lower alpha band per action x outcome condition (error bands are ±SEM across participants, n=29). Lower alpha band power was consistently higher on trials with Go actions than on trials with NoGo actions, starting already before outcome onset. **C.** BOLD signal differences between Go and NoGo actions (activation by either left or right Go actions compared to the implicit baseline in the GLM, which contains the NoGo actions; left panel) and left vs. right hand responses (right panel) at the time or responses. Response-locked dACC BOLD was significantly higher for Go than NoGo actions. **D.** BOLD signal differences between Go and NoGo actions at the time of outcomes. Outcome-locked dACC BOLD signal (and BOLD signal in other parts of cortex) was significantly lower on trials with Go than on trials with NoGo actions. ** $p < 0.01$, cluster-based permutation test.

Supplementary Note 5: Stay behavior as a function of EEG and fMRI with only the 29 participants included in EEG-fMRI analyses

We repeated the behavioral analyses reported in the main text while excluding the seven participants that were also not included in the fMRI-inspired EEG analyses in the main text: (a) two participants due to fMRI co-registration failure, which were also not included in the fMRI-only analyses; (b) four further participants who exhibited excessive residual noise in their EEG data (> 33% rejected trials) and were thus also not included in the EEG-only analyses, and finally (c) one more participant who (together with four other participants already excluded) exhibited regression weights for every regressor about ten times larger than for other participants.

When linking trial-by-trial BOLD signal in selected ROIs as well as midfrontal EEG TF power to response repetition on the next trial with the same cue, dACC BOLD signal did not significantly predict the response repetition, $\chi^2(1) = 0.524$, $p = .469$, $b = -0.013$, *95%-CI* [-0.048, 0.022], two tailed, and neither did PCC BOLD signal, $\chi^2(1) = 2.079$, $p = .149$, $b = -0.037$, *95%-CI* [-0.074, 0.001], two-tailed. However, participants in this subgroup were significantly more likely to repeat the sample action when striatal BOLD signal was high, $\chi^2(1) = 12.043$, $p < .001$, $b = 0.097$, *95%-CI* [0.048, 0.146], two-tailed, but more likely to switch when vmPFC BOLD was high, $\chi^2(1) = 13.170$, $p < .001$, $b = -0.075$, *95%-CI* [-0.112, -0.038], two-tailed.

When linking trial-by-trial midfrontal EEG TF power to response repetition on the next trial with the same cue, participants in this subgroup were more likely to repeat the same response when beta power was high, $\chi^2(1) = 3.502$, $p < .001$, $b = 0.012$, *95%-CI* [-0.058, 0.083], two-tailed, or when low alpha power was high, $\chi^2(1) = 8.789$, $p = .003$, $b = 0.014$, *95%-CI* [-0.073, 0.100], two-tailed, but more likely to switch to another response when theta power was high, $\chi^2(1) = 4.812$, $p = .028$, $b = -0.09$, *95%-CI* [-0.168, -0.012], two-tailed.

Supplementary Note 6: Parameter recovery analyses for model M5

We performed parameter recovery analyses to assess the identifiability of the model parameters in the winning "asymmetric pathways" model M5. We simulated 100 new data sets based on the best fitting parameters of each participant, fitted a separate model to each simulated data set (using first Laplace approximation and then hierarchical Bayesian inference), and finally averaged parameters across the 100 fitted models.

Parameter recovery was excellent for the feedback sensitivity ρ (r = .91), the baseline learning rate ε_0 (r = .98), the Go bias b (r > .99), and the Pavlovian response bias π (r > .99), with between-participant differences in ground-truth parameters correlating at high levels (all r > .90; Supplementary Fig. 5) with between-participant differences in the recovered parameters. Note that, due to shrinkage to the mean as a consequence of hierarchical Bayesian inference, extreme parameter values tended to be shrunk to the overall group-level mean in the recovered parameters. Correlations for the learning bias parameter κ were considerably lower, though still strongly positive (r = 0.50; r = 0.51 when removing one outlier participant; Supplementary Fig. 5E). Note however that the effect of κ on learning depended on participants' baseline learning rate ε_0. When computing increased learning rates for rewarded Go actions and decreased learning rates for punished NoGo actions—the parameters that determine the effective degree of trial-by-trial learning—these learning rates were again highly correlated with the ground truth parameters ($\varepsilon_{rewarded\ Go}$: r = 0.96; $\varepsilon_{punished\ NoGo}$: r = 0.85 resp. r = 0.86 when removing one outlier participant; Supplementary Fig. 5F-G).

Further parameter recovery analyses on the models explored in Supplementary Note 8 yielded that the recovery of κ was improved (r = 0.78) when adding perseveration parameters (which themselves had recovery performances of r's > 0.99). This observation suggested that models featuring such perseveration parameters might be better suited for quantifying individual differences in the learning bias.

In sum, parameter recovery was excellent for all parameters but the learning bias κ. More relevant than recovery of κ, however, was that we could recover the effective learning rate well (combining baseline learning rate ε_0 and the learning bias κ). However, when combining the baseline learning rate ε_0 and the learning bias κ, recovery was high, as well. Note that the ability to accurately capture individual differences in biased learning is not of interest in this study, nor relevant to the imaging analyses. In fact, we used a single set of parameters (the group-level parameters) to compute trial-by-trial regressors for the EEG and fMRI analyses. This is a standard approach in model-based fMRI for two main reasons. First, it has been shown that the exact parameter values for relatively simple RL models like the ones used here have little impact on the results of fMRI analyses [1]. For the current study, of most relevance is the qualitatively differential pattern of learning updates after Go and NoGo responses [2–4], as embodied by the algorithmic specification of the model. This pattern drives the EEG and fMRI results and indeed, using a different set of parameter values, we obtain essentially identical fMRI results (see Supplementary Note 9 and Supplementary Fig. 8).

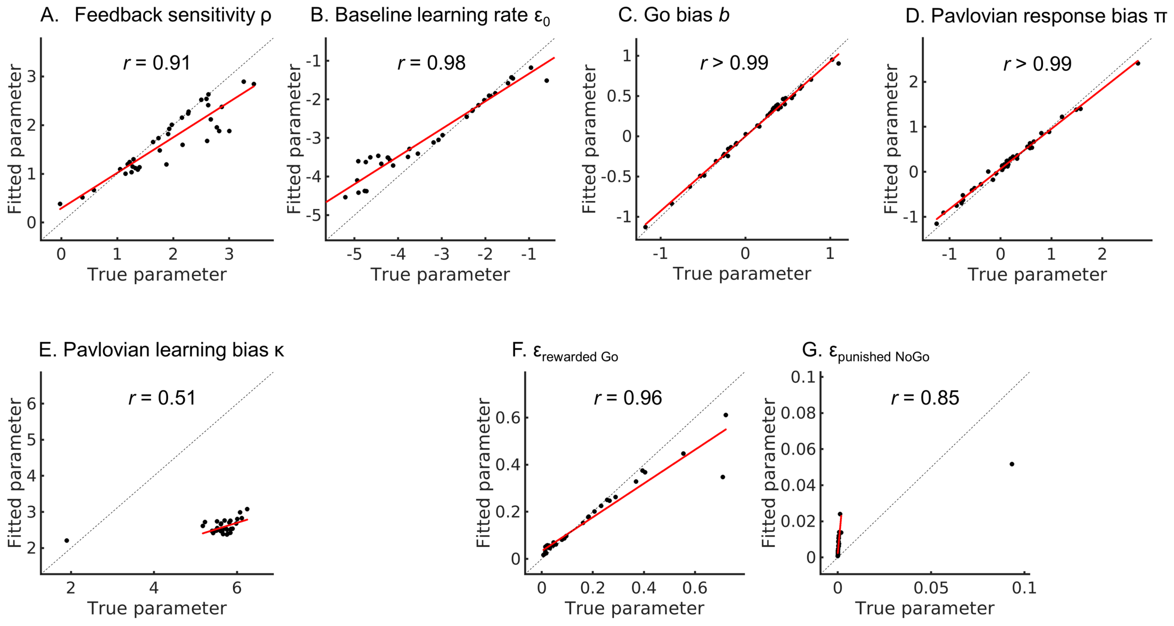

Supplementary Figure 5. Parameter recovery results for the asymmetric pathways (M5) model. The feedback sensitivity parameter ρ (**A**), the baseline learning rate ε_0 (**B**), the Go bias *b* (**C**), and the Pavlovian response bias π (**D**) all showed excellent parameter recovery, i.e., between-participants correlations of ground-truth and fitted parameters all exceeded $r > 0.90$. Parameters ρ and ε_0 are still in sampling space and thus untransformed (which means they can be negative). Dashed lines represent the identity line; red solid lines represent a linear regression line of fitted parameters regressed onto true parameters. Only recovery of the learning bias parameter κ (**E**) was not quite as good, though the correlation between ground-truth and fitted parameters was still strongly positive (r > 0.50). Note an outlier at the bottom left of κ values; the regression line was fitted without this data point. When combining the baseline learning rate ε_0 with the learning bias κ to compute the biased learning rates for rewarded Go actions $\varepsilon_{\text{rewarded Go}}$ (**F**) and punished NoGo actions $\varepsilon_{\text{punished NoGo}}$ (**G**), correlations between ground-truth and fitted parameter values were considerably higher (*r*'s > 0.86). Note again an outlier at the top right of for $\varepsilon_{\text{punished NoGo}}$ values; the regression line was fitted without this data point.

Supplementary Note 7: Simulations for asymmetric pathways and action priming model

Motivational learning biases are predicted by the *asymmetric pathways model* [5,6]: Positive PEs, elicited by rewards, lead to long-term potentiation in the striatal direct “Go” pathway (and long term depression in the indirect pathway), allowing for a particularly effective acquisition of Go actions to obtain rewards. Conversely, negative PEs, elicited by punishments, lead to long term potentiation in the NoGo pathway, impairing the unlearning of NoGo actions in face of punishments.

An alternative account has recently suggested that self-generated (Go) actions lead to preferential learning (relative to non-self-generated actions, including inaction), more generally (henceforth called “action priming model”)[7]. A self-generated action could “prime” basal ganglia circuits and lead to subsequently larger PEs and thus faster learning. The main differential prediction between these two models is how they account for the failure to learn “Go” actions to avoid punishment: In the first model, this is due to a failure to unlearn punished “NoGo” actions, while in the second model, this is due to increased unlearning of punished “Go” actions.

Here, we directly tested both models against each other. We specified an alternative model M6 [7] with two separate learning rates, one learning rate for trials where self-generated (Go) action selection should prime the processing of any following salient outcome (i.e., Go actions followed by rewards/ punishments), and one learning rate for any other action-outcome combination. In this model, equation (6) was substituted by equation (7):

$$\varepsilon = \begin{cases} \varepsilon_{salGo} & for\ any\ Go\ action\ with\ salient\ outcomes \\ \varepsilon_0 & else \end{cases} \tag{7}$$

When comparing all models M1–M6 using Bayesian model selection, M5 (the asymmetric pathways model) received highest support (model frequency: 68.15%; protected exceedance probability: 99.70%), also compared to M6 (the action priming model; model frequency: 24.19%; protected exceedance probability: 0.30%; Supplementary Fig. 6D, H). In fact, as visible in Supplementary Fig. 6E-G, the action priming did not reproduce the motivational biases in learning curves and bar plots, which constitutes a case of qualitative model falsification [2,3]. If anything, it seemed that the action priming model traded off both biases, leading to negative response biases for a majority of participants. In contrast, the asymmetric pathways model (M5) was well able to capture the qualitative patterns observed in the data (Supplementary Fig. 6A-C). We conclude that only the asymmetric pathways model is able to qualitatively reproduce core characteristics of our data.

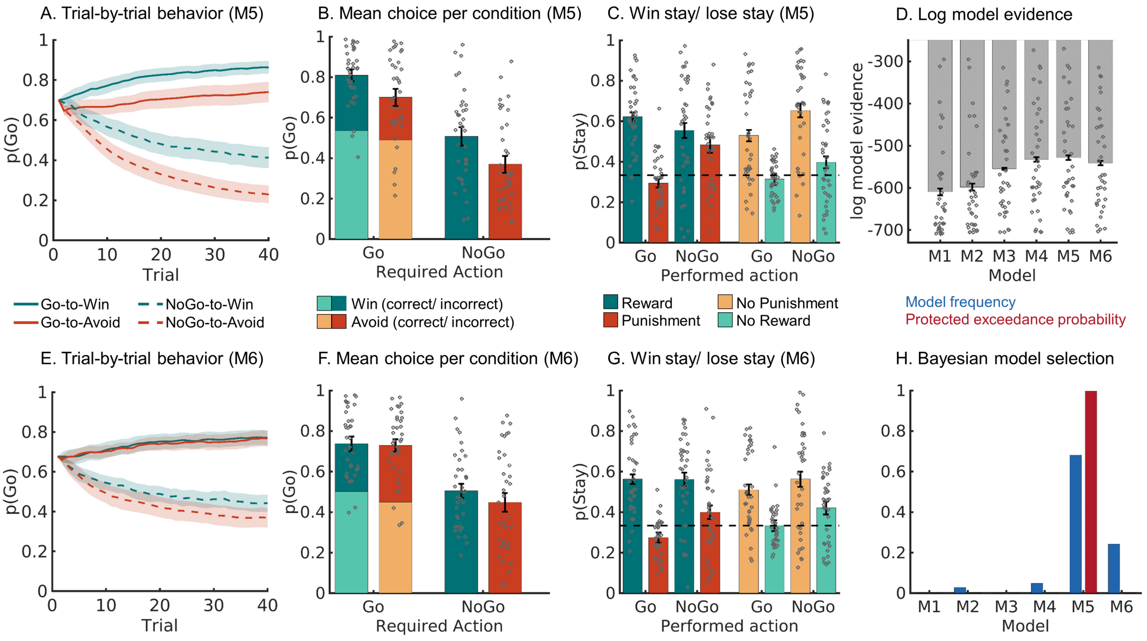

Supplementary Figure 6. Model comparison and validation of asymmetric pathways (M5) and action priming (M6) model. **(A-C**) One-step-ahead predictions using parameters (hierarchical Bayesian inference) of the winning model asymmetric pathways model (M5). **A.** Trial-by-trial proportion of Go responses (error bands are ±SEM across simulated agents, dots indicate individual participants, n=36) for Go cues (solid lines) and NoGo cues (dashed lines); **B.** Mean (error bars are ±SEM simulated agents, n=36) proportion Go responses per cue condition (points are individual participants' means); **C.** Probability of repeating a response ("stay") on the next encounter of the same cue as a function of action and outcome (error bars are ±SEM across simulated agents, n=36). The asymmetric pathways model was well able to capture core characteristics of the empirical data (see Fig. 2 in the main text). **D.** Log-model evidence favors the asymmetric pathways model (M5), even over the action priming model (M6; error bars are ±SEM across participants, n=36). **E-G.** Trial-by-trial proportion of Go responses, mean proportion Go responses, and probability of for the action priming model (M6; error bars are ±SEM across simulated agents, n=36). This model did not reproduce motivational biases (i.e., the difference between green and red lines and bars) well. **H.** Model frequency and protected exceedance probability indicate best fit for model M5 (asymmetric pathways model), in line with log model evidence.

Supplementary Note 8: Behavioral results for the perseveration model (M7), cue valence-based perseveration model (M8), and neutral outcomes reinterpretation model (M9)

While the winning model M5 reported in the main text captured learning curves and the proportion of (correct/ incorrect) Go and NoGo responses well, it did not fully capture the propensity to stay (i.e., repeat the same response to the subsequent presentation of the same cue) following different action-outcome combinations (see Fig. 2G in the main text). Specifically, M5 underestimated the overall propensity to stay and predicted a higher probability of repeating a Go response after a positive (neutral) outcome for Avoid cues, relative to the negative (neutral) outcome for Win cues. In contrast, in the data, there was no such significant difference. We thus explored three extensions of M5 that had the potential to capture this behavioral pattern. Specifically, we considered mechanisms that would make the model more likely to repeat a given response. Furthermore, any such mechanism should boost repetition of Go responses to non-rewarded Win cues particular. We hypothesized that two potential mechanisms could account for these data features, and present three new models to test these mechanisms.

As a first mechanism, we considered overall "response stickiness" or "perseveration"[8], a process that leads participants to repeat a previous response independent of the obtained outcome. This mechanism could explain participants' overall higher propensity to stay, which we tested in model M7. ***Model M7***, called "***single perseveration model***", featured the same parameters as M5 plus a perseveration parameter φ that was added as a "bonus" to the action weight $w(a_i, s_t)$ of the specific action shown on the last occurrence of the respective cue[8]:

$$w(a_i, s_t) = \begin{cases} w(a_i, s_t) + \varphi & \textit{if last action to same cue was } a_i \\ w(a_i, s_t) & \textit{else} \end{cases} \tag{8}$$

In M7 equation 7 in the main manuscript was replaced by equation 8 above, such that parameter φ captured the propensity to repeat the action from the last time this cue was presented.

However, to account for the fact that staying was not different and numerically even higher for a non-rewarded Go response (to a Win cue), relative to a non-punished Go response to an Avoid cue, we tested whether separate perseveration parameters for Win and Avoid cues could capture this behavioral difference (M8), as such a pattern of results could result from an overall higher propensity to stay for Win cues. **This "cue valence-dependent perseveration model" (M8)**, contained two separate perseveration parameters, one for Win cues φ_{WIN}, and one for Avoid cues φ_{AVOID}. The respective perseveration parameter was added to the action weight $w(a_i, s_t)$ of the specific action shown on the last occurrence of respective the cue:

$$w(a_i, s_t) = \begin{cases} w(a_i, s_t) + \varphi_{WIN} & \textit{if Win cue and last action to same cue was } a_i \\ w(a_i, s_t) + \varphi_{AVOID} & \textit{if Avoid cue and last action to same cue was } a_i \\ w(a_i, s_t) & \textit{else} \end{cases} \tag{9}$$

In M8, equation 7 in the main manuscript was replaced by equation 9 above, such that parameter φ_{WIN} and φ_{AVOID} captured the propensity to repeat the action from the last time this cue was presented, separately for Win and Avoid cues.

As an alternative mechanism that could potentially capture the p(stay) pattern in the data, we considered the possibility that participants might "re-interpret" neutral outcomes in line with the cue valence: although a non-reward after a Win cue constitutes negative feedback, the positive cue valence might "overshadow" this feedback and give participants the impression that they received a reward. Similarly, a non-punishment after an Avoid cue constitutes positive feedback, but the negative cue valence might overshadow this feedback and give participants the impression that they received a punishment.

Following this idea, lastly, we considered **M9,** called the "**neutral outcome reinterpretation model**", which featured a single perseveration parameter φ as in equation (8), but in addition replaced neutral outcomes (coded as zero) with what we term the "effective reward" r_{EFF}, which allows the neutral outcome to take on a value in the direction of the cue valence $V(s)$. The degree to which this happens is scaled by the parameter η:

$$r_{EFF} = \begin{cases} \mathrm{V(s)} * \eta & if\ r = 0 \\ r & else \end{cases} \qquad (10)$$

We subsequently used r_{EFF} for computing prediction errors. Thus **M9** adds equation 10 to model **M7**. Note that for $\eta = 0$, neutral outcomes stay at zero and M9 becomes equivalent to M7.

Bayesian model comparison across the winning original model M5 and these three new models yielded highest model evidence for M8, followed by M9 (model frequency: M5: 3%, M7: 0%, M8: 62%, M9: 35%; protected exceedance probability: M5: 0%, M7: 0% M8: 95%, M9: 5%). All three models performed better than the original winning model M5 (Supplementary Fig. 7, bottom row). Simulations showed that the best fitting model M8 (with separate perseveration rates for Win and Avoid cues) indeed better captured the propensity to stay on neutral trials, though this came at the cost of a general overestimation of staying after punished responses (which hold similarly for M7 and M9; see Supplementary Fig. 7, third row). More importantly, however, this model drastically underestimated the crucial pattern of behavior under study here, namely the propensity of incorrect, bias-driven Go responses to Win cues (see Supplementary Fig. 7, second row, dark green part of bars).

In sum, the three additional models provided a better quantitative fit to the data compared to the winning model M5 reported in the main text. Also, these additional models predicted the propensity more accurately than the base models did. However, their qualitative fit (i.e. the ability to capture relevant aspects of the data) was worse: These additional models systematically underestimated the proportion of incorrect Go responses (Supplementary Fig. 7). Furthermore, although the predicted patterns of the propensity to stay matched the data more closely than M5, these predicted patterns still mis-matched some aspects of the data, particularly now over-estimating the tendency to stay following a punishment. Taken together, these models could capture certain qualitative patterns in the data, but not others, which is a core feature of computational modelling, which by definition constitutes a data reduction procedure that necessarily loses some details of the data. In terms of qualitative model validation/ falsification[2,3], M5 and M8/M9 capture different qualitative features of the data, but no model captured all features well.

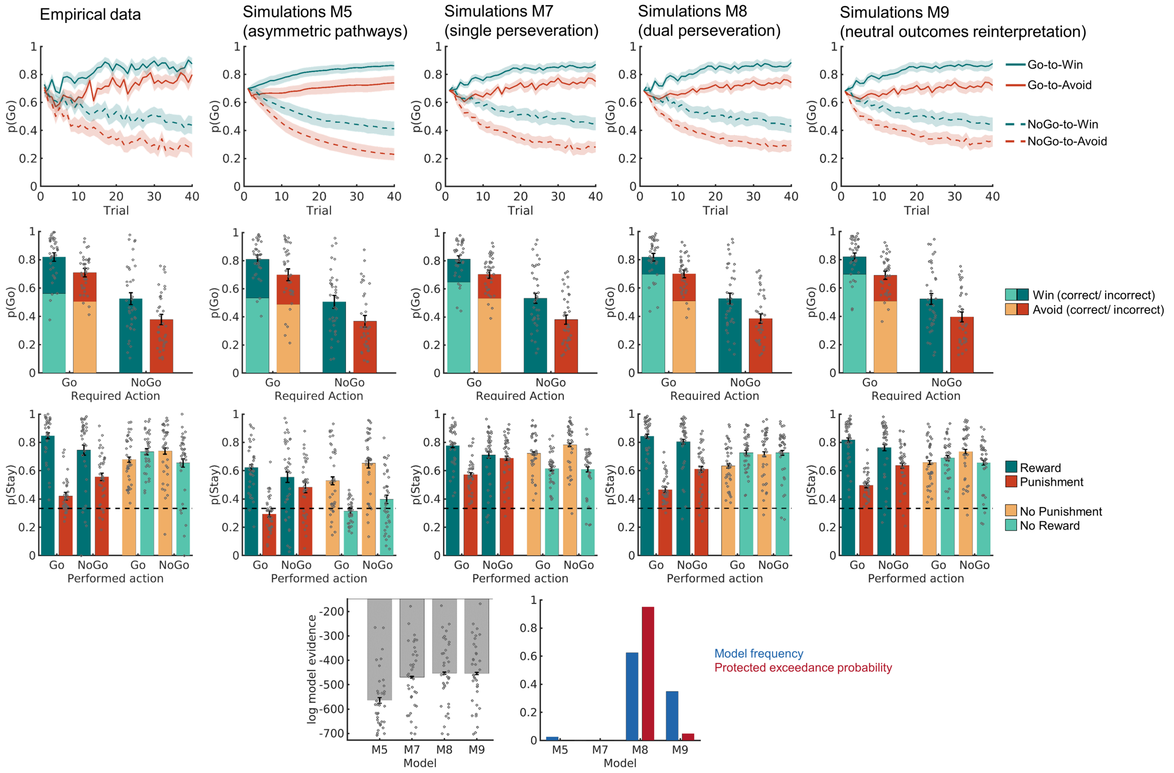

Supplementary Figure 7. Model comparison and validation of the single perseveration (M7), dual perseveration (M8) and cue valence-based outcome reinterpretation models. **First row.** Trial-by-trial proportion of Go responses (error bands are ±SEM across participants, n=36) for Go cues (solid lines) and NoGo cues (dashed lines). **Second row.** Mean (error bars are ±SEM across participants, n=36) proportion Go responses per cue condition (points are individual participants' means). **Third row.** Probability to repeat a response ("stay") on the next encounter of the same cue as a function of action and outcome (error bars are ±SEM across participants, n=36). **Fourth row.** Log-model evidence, model frequency, and protected exceedance probability all favored the dual perseveration model (M8) over the other models (error bars are ±SEM across participants, n=36). In sum, the additional models M7-9 provided a better quantitative fit to the data compared to the asymmetric pathways model M5 reported in the main text. They also predicted the propensity of staying overall more accurately than M5. However, these additional models all overestimated the proportion of incorrect Go responses. Furthermore, although the predicted patterns of the propensity of staying mimicked the data more closely than M5, these predicted patterns still mismatched some aspects of the empirical data. Taken together, these models could capture certain qualitative patterns in the data, but not others, which was expectable given the data reduction that comes with fitting a learning model with few parameters only.

Supplementary Note 9: Neural results based on prediction-errors from the cue valence-based perseveration model (M8) and neutral outcomes reinterpretation model (M9)

To confirm that neural correlates of biased prediction-error updating were not altered under these alternative model specifications, we repeated the model-based fMRI analyses for both the cue valence-dependent perseveration model M8 and the neutral outcomes interpretation model M9. In summary, the results are effectively unchanged, as we present in more detail below.

Notably, M8 does not make different predictions about trial-by-trial learning updates; the only difference to M5 consisted in slightly different best fitting parameter estimates for ε and κ (leading a slightly different BOLD regressors. Neural correlates of learning typically reflect the qualitative learning pattern, which is the same for M5 and M8, but are hardly sensitive to the exact parameter values [1]. Indeed, when repeating the fMRI analyses with those different parameter values, we found almost identical results, with significant encoding of both PE_{STD} and PE_{DIF} in striatum, dACC, pgACC, PCC, left motor cortex, left ITG, and V1 (Supplementary Fig. 8A, B). The only exception was the cluster in dACC, which under M8 was not significant at a whole-brain level, but significant when using small-volume correction with an anatomical ACC mask (from the Harvard-Oxford Atlas), warranted by our a-priori hypotheses based on previous literature[9].

When we repeated our fMRI analyses with learning updates predicted by M9, we again found significant encoding of both PE_{STD} and PE_{DIF} in striatum, dACC, pgACC, PCC, left motor cortex, left ITG, and V1 (Supplementary Fig. 8C). However, the pgACC cluster was much larger and extended into the vmPFC. Similarly, the PCC cluster was much larger. In addition, BOLD signal in left inferior frontal gyrus and in multiple clusters in superior and inferior lateral occipital cortex encoded both PE_{STD} and PE_{DIF} significantly. Using trial-by-trial BOLD signal from the extended vmPFC and PCC clusters identified with M9 regressors to predict midfrontal EEG power, we obtained results that were highly similar to the results for the pgACC and PCC clusters identified with M5 regressors.

In sum, model-based fMRI analyses based on PEs derived from M8 and M9 replicated the findings based on M5 reported in the main text. In addition, M9 led to larger clusters in vmPFC and PCC, tentatively suggesting that these regions might potentially contribute to "reinterpreting" neutral outcomes in light of the previously presented cue valence (see also Fig. 2 in the main text).

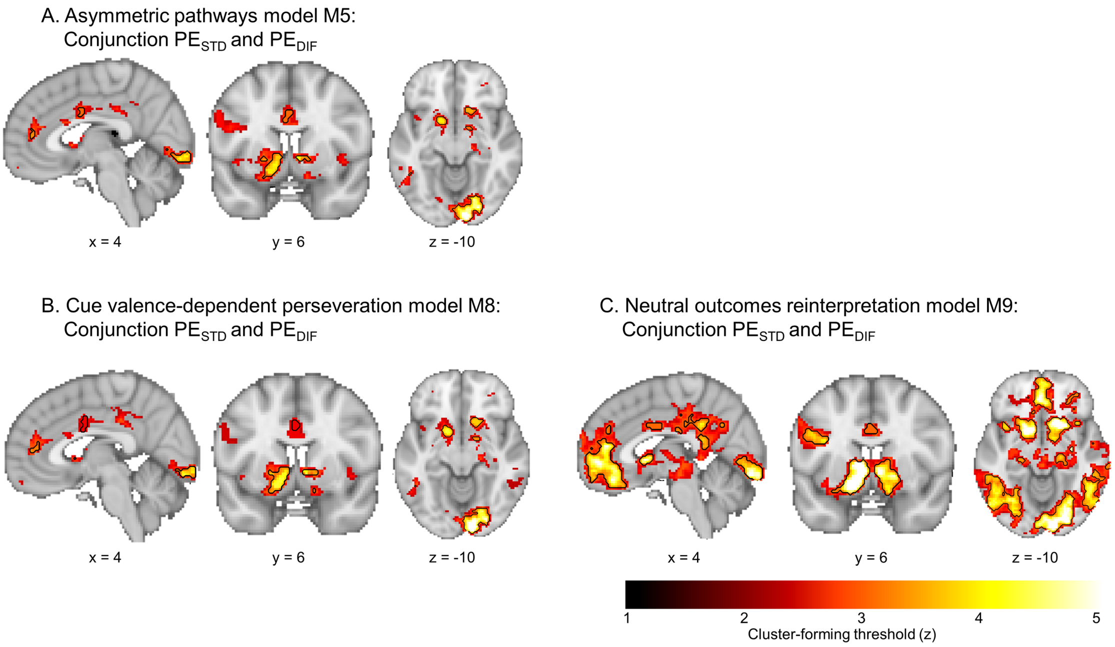

Supplementary Figure 8. BOLD correlates of biased prediction errors as predicted by the asymmetric pathways model (M5), the cue valence-dependent perseveration model (M8) and the neutral outcomes reinterpretation model (M9). **A**. Regions encoding both the standard PE term and the difference term to biased PEs (conjunction) as predicted from the asymmetric pathways model (M5) at different cluster-forming thresholds ($1 < z < 5$, color coding; opacity constant; replotted from Fig. 3C main text). Clusters significant at a threshold of $z > 3.1$ are surrounded by black edges. This is a version of Fig. 3C reprinted with a color scheme consistent with the other two panels. **B**. Regions encoding both the standard PE term and the difference term to biased PEs (conjunction) as predicted from the cue valence-dependent perseveration model (M8) at different cluster-forming thresholds ($1 < z < 5$, color coding; opacity constant). Clusters significant at a threshold of $z > 3.1$ are surrounded by black edges. In line with correlates of biased PEs as predicted by M5, BOLD signal in bilateral striatum, dACC (small-volume corrected), pgACC, PCC, left motor cortex, left inferior temporal gyrus, and primary visual cortex was significantly better explained by biased learning than by standard learning. This finding was not surprising given that adding perseveration to the model did not change the learning mechanism, but only led to slightly different best fitting parameter values. **C.** Regions encoding both the standard PE term and the difference term to biased PEs (conjunction) as predicted from the neutral outcomes reinterpretation model (M9). In addition to the regions in which BOLD signal was significantly better explained by biased than standard PEs as derived from M5 and M8, biased PEs derived from M9 also explained BOLD signal in vmPFC (larger cluster than M5), PCC (larger cluster than M5), left inferior frontal gyrus and multiple clusters in superior and inferior lateral occipital cortex significantly better than standard PEs. These results tentatively suggested that vmPFC, PCC, and these other occipital regions might implement an additional mechanism besides biased learning which encodes the cue valence also at the time of the outcome, biasing the processing of neutral outcomes.

Supplementary Note 10: Illustration of biased and standard prediction error regressors for a representative example participant

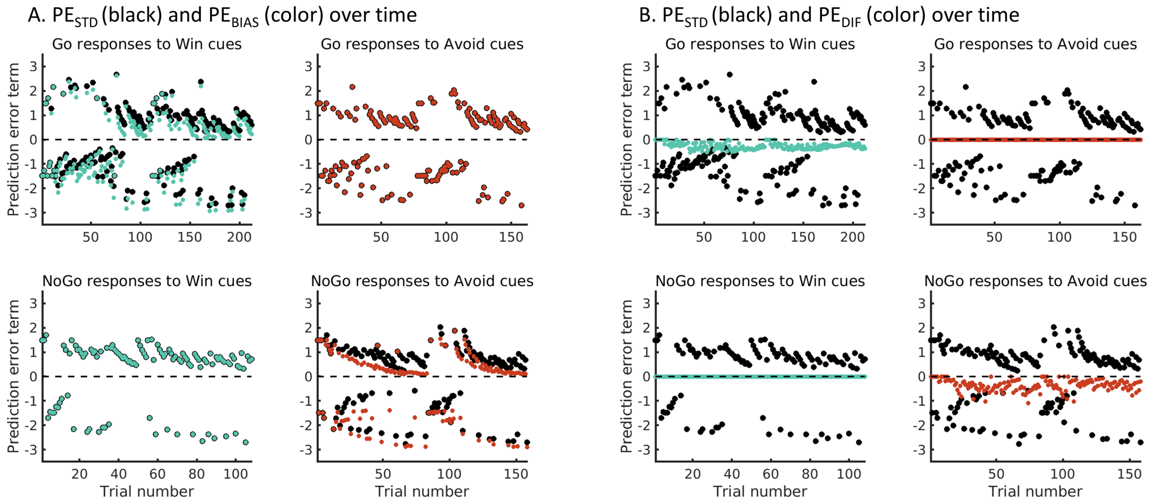

Supplementary Figure 9. Illustration of biased and standard learning for a representative example participant. **(A)** Prediction errors according to the standard Q-learning model M1 (PE_{STD}; black dots) and according to the winning model M5 implementing biased learning (PE_{BIAS}; colored dots). In M5, motivational biases partially arise through biased learning: Participants learn more readily that an action has caused a reward, and are reluctant to learn that inaction has led to a punishment. For each cue, the values of each of the three possible actions (Go_{LEFT}, Go_{RIGHT}, NoGo) are learnt independently, and prediction errors are calculated relative to the value of the chosen action. The learning bias acts such that the effective learning rate is increased when a reward follows any Go response, and decreased when a punishment follows a NoGo response (see equation 5 in the main manuscript). Hence, for Win cues, action values for Go responses (but not NoGo responses) will be affected by the learning bias and approach the positive asymptote more quickly compared to standard learning, leading to faster decay of positive prediction errors. At the same time, negative outcomes will remain surprising and elicit larger prediction errors compared to standard learning. Hence, model predictions diverge for prediction errors after Go responses to Win cues, but not after NoGo responses to Win cues (colored dots are on top of black dots). Vice versa, for Avoid cues, action values for NoGo responses (but not Go responses) are affected by the learning bias and approach the negative asymptote more slowly compared to standard learning (with negative prediction errors remaining high) as participants are reluctant to take punishments after NoGo responses into account. At the same time, ignoring punishments leads to a faster approach of positive action values to the positive asymptote (and a faster decay of positive prediction errors) compared to standard learning. Model predictions diverge for prediction errors after NoGo responses to Avoid cues, but not after Go responses to Avoid cues (colored dots are on top of black dots). (B) To assess evidence for biased learning despite this high multicollinearity, we decomposed PE_{BIAS} into PE_{STD} (black dots) plus a difference term $PE_{DIF} = PE_{BIAS} - PE_{STD}$ (colored dots). Note that PE_{DIF} is always zero after NoGo responses to Win cues and Go responses to Avoid cues as both M1 and M5 make identical predictions for these action values. In contrast, for Go responses to Win cues and NoGo responses to Avoid cues, the PE_{DIF} term is always negative because, in both cases, positive action values approach the positive asymptote more quickly (such that positive prediction errors decay more quickly) compared to standard learning, and negative action values approach the negatively asymptote more slowly (and thus negative prediction errors remain high) compared to standard learning.

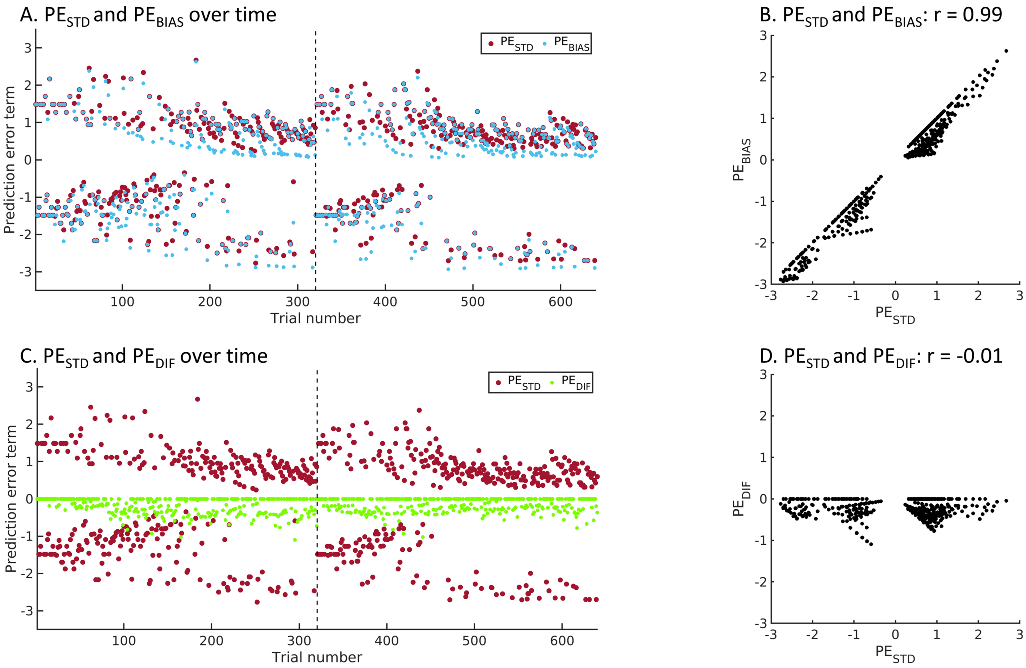

Supplementary Figure 10. Illustration of prediction error regressor decomposition for a representative example participant. **(A)** Prediction errors according to the standard Q-learning model M1 (PE_{STD}; larger red dots) and according to the winning model M5 implementing biased learning with more learning from rewarded Go responses and less learning from punished NoGo responses (PE_{BIAS}; smaller blue dots; blue dots with a red edge reflect trials on which both models make identical predictions). Both prediction error types have a highly similar profile. The key difference between them is an overall downwards shift of PE_{BIAS} compared to PE_{STD}, with positive PE_{BIAS} approaching zero more quickly than positive PE_{STD}, while negative PE_{BIAS} remain more negative compared to negative PE_{STD}. Note that, after trial 320, session 2 starts (vertical dashed line), featuring new cues. **(B)** The prediction errors from both models are highly correlated (mean across participants: r = 0.99, range 0.96–0.99), implicating that, when entered together into a multiple linear regression, both regressors would share most of their variance, which would be attributed to neither of them. **(C)** To assess evidence for biased learning despite this high multicollinearity, we decomposed PE_{BIAS} into PE_{STD} plus a difference term $PE_{DIF} = PE_{BIAS} - PE_{STD}$. PE_{STD} and PE_{DIF} show markedly different profiles, with PE_{DIF} being zero for trials on which both PE_{STD} and PE_{BIAS} make identical predictions, and being negative otherwise (reflecting the relatively faster decay of positive $\mathrm{PE}_{\mathrm{BIAS}}$ and slower decay of negative PE_{BIAS}). **(D)** Both PE_{STD} and PE_{DIF} are much less correlated (mean across participants: r = -0.02, range -0.07–0.09), making it possible to enter them in the same multiple linear regression and test whether $\mathrm{PE}_{\mathrm{DIF}}$ predicts variance in BOLD signal above and beyond PE_{STD}.

Supplementary Note 11: Masks for fMRI analyses

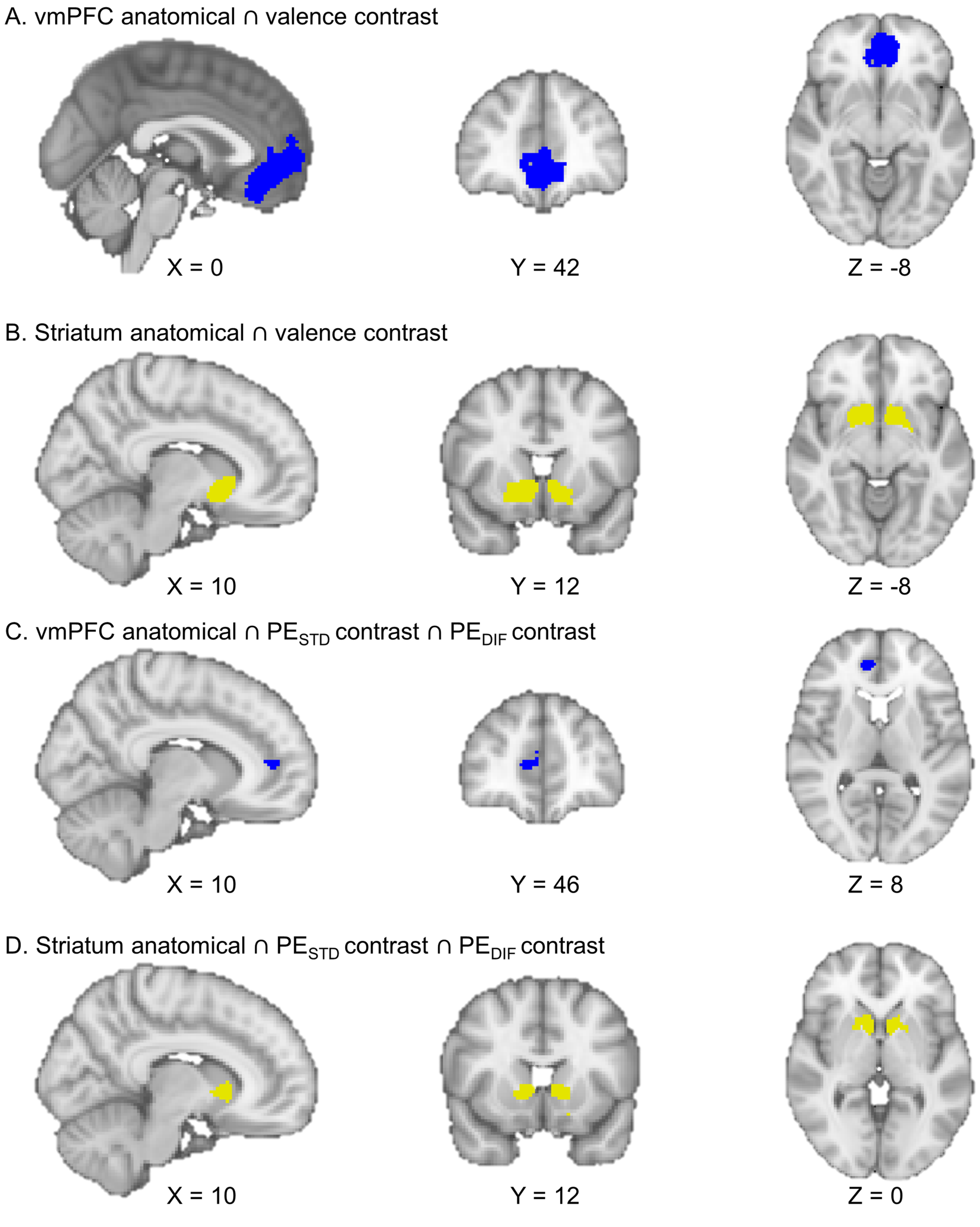

Supplementary Figure 11. Conjunctions of anatomical masks with functional contrasts from fMRI GLM analyses used for fMRI-informed EEG analyses. Anatomical masks were based on the Harvard-Oxford Atlas. Functional contrasts involve outcome valence and conjunction of PE_{STD} and PE_{DIF}. **A.** vmPFC outcome valence contrast (dark blue, conjunction of frontal pole, frontal medial cortex, and paracingulate gyrus). **B.** striatum outcome valence contrast (yellow, conjunction of bilateral nucleus accumbens, caudate, and putamen). **C.** vmPFC PE_{STD} ∩ PE_{DIF} contrast (dark blue, results in a cluster in pgACC). **D.** striatum PE_{STD} ∩ PE_{DIF} contrast (yellow). All anatomical masks were extracted from the probabilistic Harvard-Oxford Atlas, thresholded at 10%. Note that images are in radiological orientation (i.e., left brain hemisphere presented on the right and vice versa).

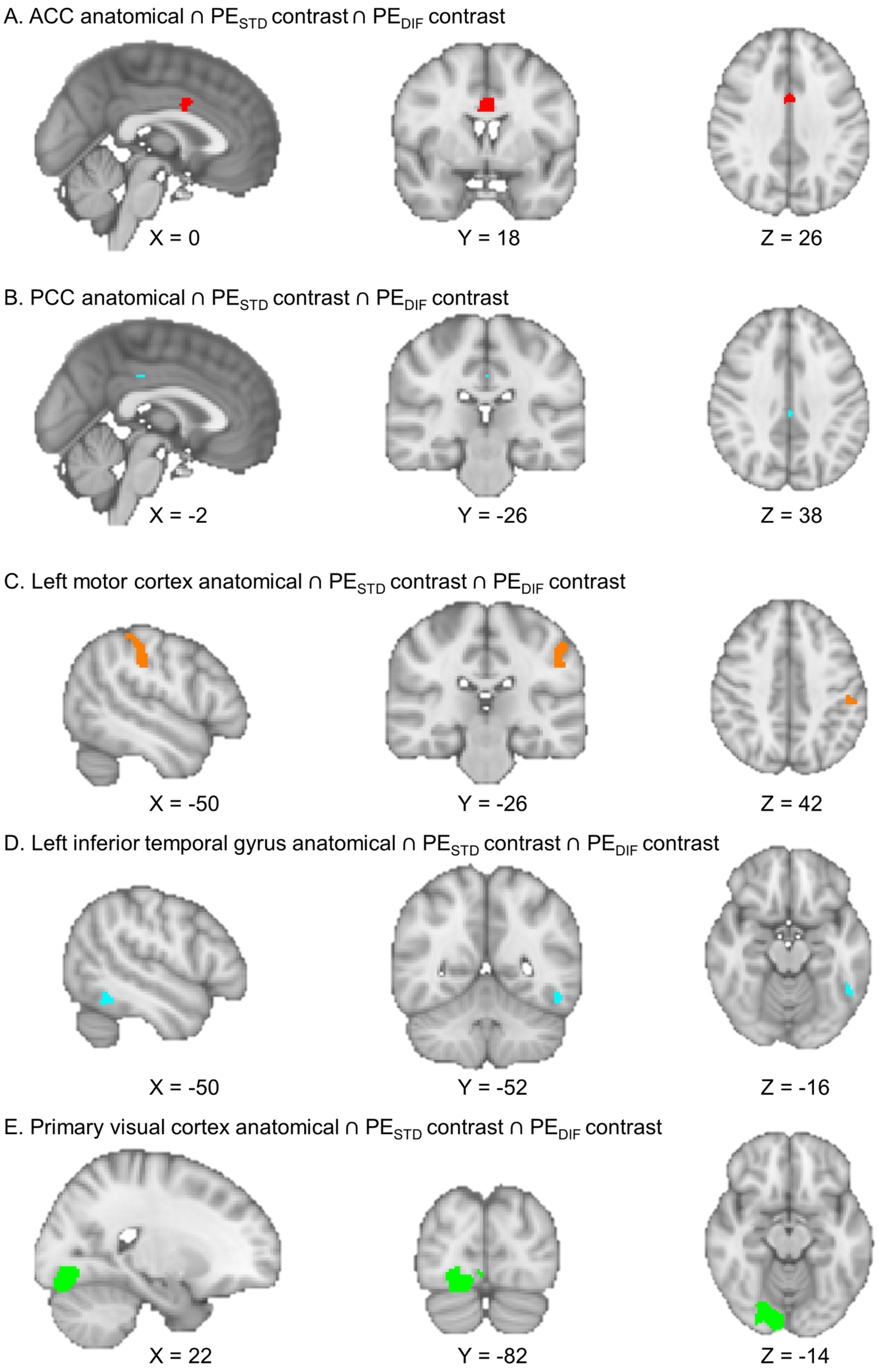

Supplementary Figure 12. Conjunctions of anatomical masks with functional contrasts from fMRI GLM analyses used for fMRI-informed EEG analyses: **A.** AAC PE_{STD} ∩ PE_{DIF} contrast (red, cingulate gyrus, anterior division, resulting in a cluster in dACC; **B.** PCC PE_{STD} ∩ PE_{DIF} contrast (light blue, cingulate gyrus, posterior division); **C.** Left motor cortex PE_{STD} ∩ PE_{DIF} contrast (orange, conjunction of precentral and postcentral gyrus). **D.** Left inferior temporal gyrus PE_{STD} ∩ PE_{DIF} contrast (turquoise, conjunction of inferior temporal gyrus, posterior division, and inferior temporal gyrus, temporooccipital part). **E.** Primary visual cortex PE_{STD} ∩ PE_{DIF} contrast (green, conjunction of lingual gyrus, occipital fusiform gyrus, occipital pole). All anatomical masks were extracted from the probabilistic Harvard-Oxford Atlas, thresholded at 10%. Note that images are in radiological orientation (i.e., left brain hemisphere presented on the right and vice versa).

Supplementary Note 12: EEG time-frequency results after ERPs were removed

Given that differences in theta power between positive and negative outcomes as well as differences in lower alpha band power after Go and NoGo responses occurred quite soon after cue onset, we aimed to test whether these effects reflected differences in evoked rather than induced activity. For this purpose, we removed evoked components from our data by computing the ERP for each of the eight conditions (action x outcome) for each participant and then subtracting the condition-specific ERP from the trial-by-trial data [10]. Only afterwards, we performed time-frequency decomposition.

In line with the results reported in the main text, power was higher for negative compared to positive outcomes in the theta band (p =.018, two-tailed, driven by cluster at 225–475 ms; Supplementary Fig. 11A, B), but higher for positive than negative outcomes in the beta band ($p < .001$, two-tailed, driven by cluster at 0–1250 ms; Supplementary Fig. 11A, C). Notably, unlike the results reported in the main text (Fig. 4A), the cluster of high power for negative compared to positive outcomes was constrained to the theta range, and did not extend further into the delta range (Supplementary Fig. 11A).

When using the trial-by-trial PEs (both the standard PE and the difference term to a biased PE) as predictors in a multiple linear regression at each time-frequency-channel bin while controlling for PE valence, delta power encoded PE_{STD} positively, though not significantly (p = .198, two-tailed). However, at a later time point around outcome offset, delta (and theta) power in fact correlated negatively with PE_{STD} (575–800 ms, p = .002, two-tailed; Supplementary Fig. 11E). The correlation between delta and the PE_{DIF} term was still positive, but not significant (p = .228, two-tailed; Supplementary Fig. 11F). Similarly, the correlation of the PE_{BIAS} term with delta power was positive, but not significant (p = .084, two-tailed; Supplementary Fig. 11D).

Regarding beta power, there was a positive, though non-significant correlation of beta power with PE_{STD} (p = .096, two-tailed; Supplementary Fig. 11E). There was again a significantly negative correlation of beta power with PE_{DIF} (425–875 ms, $p < .001$, two-tailed; Supplementary Fig. 11F). Likewise, beta power correlated significantly negatively with PE_{BIAS} (450–800 ms, p = .018, two-tailed; Supplementary Fig. 11D), driven by the correlation with PE_{DIF}.

In sum, after subtracting the condition-wise ERP from each trial before time-frequency decomposition, supposedly removing the phase-locked aspect of power, both beta and theta still encoded PE valence. However, the encoding of PE magnitude by delta power was attenuated and not significant any more.

This reduction in magnitude encoding might occur of several reasons. Firstly, it might be that this correlation in the delta range was in fact (partly) reflecting correlations with phase-locked, i.e., evoked activity (ERPs), especially in the N2 (FPN)/ P3 (RewP) time range (see Supplementary Note 11 and Supplementary Fig. 12)[11–20]. Nonetheless, a positively correlation between delta power and biased PEs was still visible in Supplementary Fig. 11D, suggesting that at least part of the signal encoding biased PEs was not phase-locked. Secondly, it might be that the removal of the condition-wise ERPs has introduced additional noise in the data, attenuating any true correlation. Thirdly, there was a negative correlation between PE_{STD} and theta/ delta power at later time points which was visible, though not significant in the results reported in the main text (Fig. 4D). Subtraction of an ERP-like template acts like a high-pass filter. High-pass filtering at relatively high cut-offs (> 0.5 Hz) can artificially postpone or induce effects at later points [21]. It is possible that in this case, ERP subtraction attenuated a positive correlation in the theta/ delta range, but enhanced a later negative correlation.

Taken together, it is possible that part of the PE magnitude encoding in the theta/ delta range is due to correlations with the phase-locked (ERP) signal. However, this finding did not compromise the

conclusion that overall, theta/delta power seemed to be more strongly associated with the PE_{BIAS} term than the PE_{STD} term. Our primary goal was not to pinpoint the precise nature of electrophysiological correlates of biased learning, but rather test the relative temporal order of when different regions exhibiting biased learning signals become active.

Finally, we tested whether after ERP subtraction, low alpha (and beta power) still encoded the previously performed action. When testing for differences in broadband power after Go and NoGo responses, power was indeed significantly different between conditions, driven by clusters in beta band (p = 0.002, two-tailed, 0.125–625 ms; p = 0.052, two-tailed, 700–1000 ms, 23–29 Hz) and theta/ low alpha band (p = 0.024, two-tailed, 575–1000 ms, 5–9 Hz; p = 0.056, two-tailed, 0–225 ms, 6–11 Hz). For power before outcome onset, there were again broadband differences between Go and NoGo (p = 0.002, two-tailed, -1000 – +225 ms, 1–33 Hz), but note that there was no ERP subtracted before outcome onset. We thus conclude that the differences between Go and NoGo responses were attributable to differences in induced rather than evoked activity.

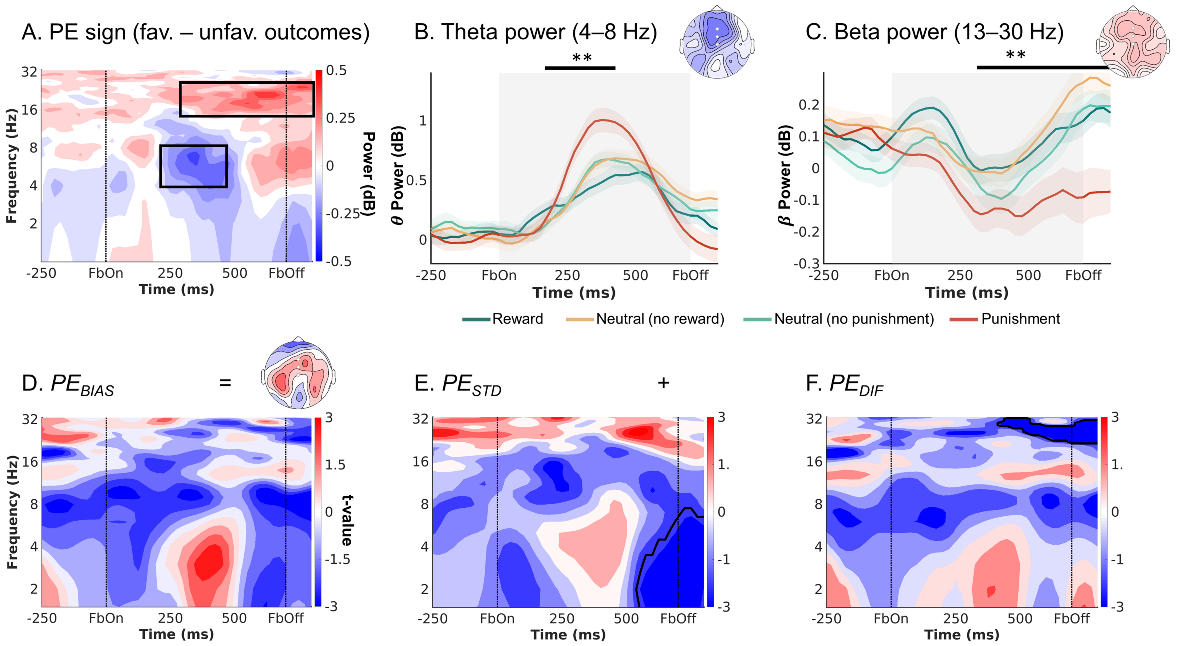

Supplementary Figure 13. EEG time-frequency power over midfrontal electrodes (Fz/ FCz/ Cz) after the (action x outcome) condition-wise ERPs has been removed. **A.** Time-frequency plot (logarithmic y-axis) displaying high theta (4–8 Hz) power for negative outcomes and higher beta power (16–32 Hz) for positive outcomes. **B.** Theta power transiently increases for any outcome, but more so for negative outcomes (especially punishments) around 225–475 ms (p =.018, two-tailed) after feedback onset. **C.** Beta was higher for positive than negative outcomes (especially punishments) over a long time period around 300–1,250 ms (p < .001, two-tailed) after feedback onset. **D-F.** Correlations between midfrontal EEG power and trial-by-trial PEs. Solid black lines indicate clusters above threshold. There still was a visible positive correlation between biased PEs and midfrontal delta power, but this correlation was not significant (**D**). The correlation of delta with the standard PEs (**E**) was also positive, though not significant (p = .198, two-tailed); in fact, at a later time point around stimulus offset, delta power correlated significantly negatively with standard PEs (575–800 ms, p = .002, two-tailed). The difference term to biased PEs (**F**) also correlated positively, though not significantly with delta power (p = .228, two-tailed). Beta power encoded the difference term (p < .001, two-tailed; panel D) and biased PEs themselves (p = .018, two-tailed; panel F). ** p < 0.01, cluster-based permutation tests.

Supplementary Note 13: ERPs as a function of action and outcome

In addition to the induced activity in time-frequency power reported in the main text, we also analyzed the data in the time domain to test for differences in evoked activity. These analyses were particularly motivated given that differences in time-frequency power between positive and negative outcomes (theta/delta range) and after Go and NoGo responses (lower alpha/ theta range) occurred soon after outcome onset, warranting the assumption that differences might also occur in evoked activity. A large range of previous research has reported a modulation of evoked potentials by outcome valence in form of the feedback-reduced negativity [14–20,22], i.e., a stronger N2 component for negative compared to positive outcomes around ~ 250 post-cue over midfrontal electrodes, recently also characterized as rather constituting a reward positivity (RewP) [14]. Also, some studies have reported a modulation of the P3 by outcome valence, which has been attributed to outcome magnitude or salience rather than valence [17,18,20,23].

Similar to the analysis of time frequency power, we sorted trials into the eight conditions spanned by the performed action (Go/ NoGo) and the obtained outcome (reward/ no reward/ no punishment/ punishment), computed the average ERP for each condition per participant, and tested for differences between positive (reward/ no punishment) and negative (no reward/ punishment) outcomes as well as conditions of relative stronger (rewarded Go and punished Go) vs. relatively weaker learning (rewarded NoGo and punished NoGo). We used cluster-based permutation tests on the average signal over midfrontal electrodes (Fz/ FCz/ Cz) in the time range of 0–700 ms after outcome onset (where evoked potentials visible in condition-averaged plot).

First, midfrontal ERPs were significantly different between positive and negative outcomes, driven by two separate clusters of differences above threshold (Cluster 1: around 246 – 294 ms, p = .034, two-tailed; Cluster 2: around 344–414 ms, p =.004, two-tailed; Supplementary Fig. 12A, C). The first cluster the classical feedback-related negativity, i.e., a stronger N2 component for negative compared to positive outcomes. The second cluster reflected weaker P3 component for negative compared to positive outcomes, similar the reward positivity reported before. In fact, the N3 was rather absent for negative outcomes (Supplementary Fig. 13). Both effects were clearly focused on midfrontal electrodes. These findings replicate previous findings of outcome valence modulating N2 (feedback-related negativity) and P3 components, and complement our time-frequency findings of theta and beta power reflecting outcome valence.

Second, when contrasting trials with Go vs. NoGo responses, no significant difference was observed (p = .358, two-tailed; Supplementary Fig. 12D). Visual inspection of the topoplot yielded that, if anything, differences emerged over right occipital electrodes. If one performed a test over those right occipital electrodes (O2, 04, PO4; Supplementary Fig. 12F; note that this procedure constitutes double-dipping because the test was informed by first looking at the data), this test would have yielded significant results (p = .016, two-tailed) driven by cluster around 423–466 ms, reflecting a slightly larger P3 after Go than NoGo responses (Supplementary Fig. 12E). This finding appears to be the strongest (if any) difference in amplitude after outcome onset between Go and NoGo actions. Given that this difference was not hypothesized and occurred far away from our a-priori selected channels of interest, we are careful not to over-interpret those differences.

Third, contrasting trials with positive and negative at the same right occipital electrodes yielded a significant difference, driven by clusters around 46–103 ms (p = 0.034, two-tailed), 141–255 ms (p = .002, two-tailed), and 519 – 580 ms (p = .034, two-tailed). Most notably, the P1 amplitude was much larger for positive than negative outcomes (Supplementary Fig. 12B). However, given that these differences were not hypothesized and occurred far away from our a-priori selected channels of interest, we are careful not to over-interpret those differences.

Taken together, we found a bigger midfrontal N2/ FRN for negative compared to positive outcomes, and a bigger midfrontal P3/ RewP for positive compared to negative outcomes, in line with a vast literature of previous findings [14–20,22,23]. Midfrontal voltage did not significantly differ after Go or NoGo responses. If anything, differences after Go and NoGo responses were maximal over right occipital electrodes, with a larger P3 after Go than after NoGo responses. Signal at these channels also differed between positive and negative outcomes, most notably with a bigger P1 after positive than negative outcomes. In sum, we replicate classical reward learning ERP effects, which shows that the motivational Go/NoGo learning task taps into reward learning processes reported before, but these processes appeared to be unaffected by the previously performed action.

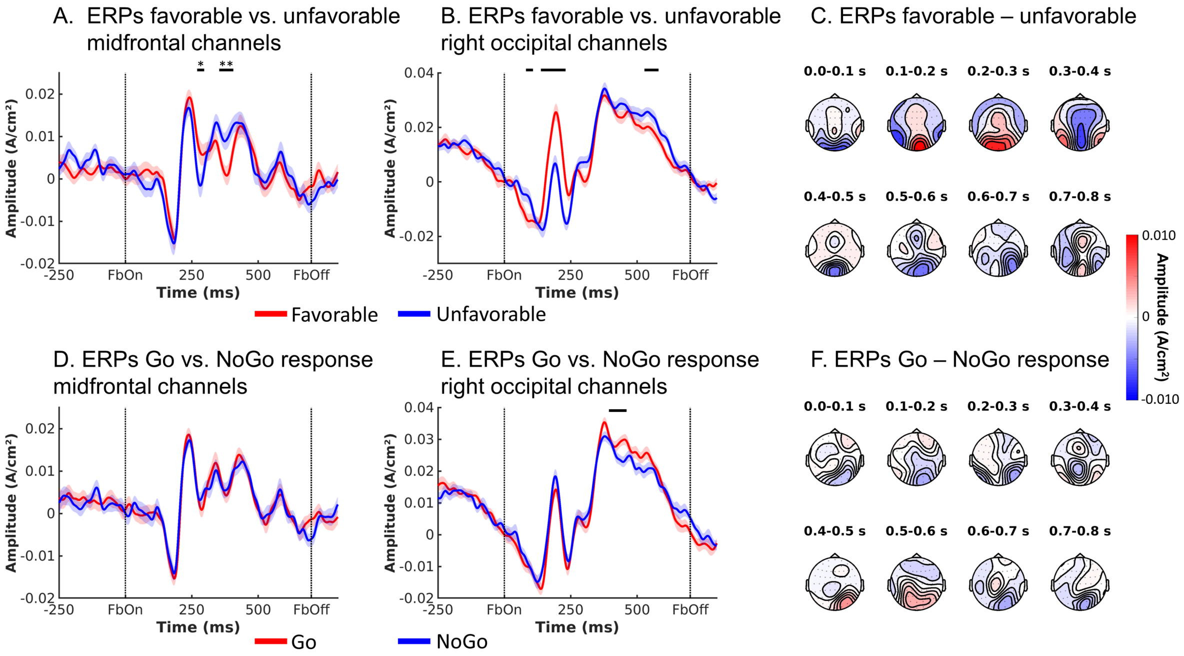

Supplementary Figure 14. ERPs reflecting outcome valence and performed action. **A.** Voltage (error bands are ±SEM across participants, n=32) over midfrontal electrodes (Fz/FCz/Cz) was lower for negative than positive outcomes around 246–294 ms (p = .034, two-tailed; stronger N2, FRN) and higher for positive than negative outcomes around 344–414 ms (p =.004, two-tailed; stronger P3/ RewP). **B.** Over right occipital electrodes, the P1 was bigger for positive than negative outcomes (141–255 ms; p = .002, two-tailed). **C.** Topoplots of difference in voltage between trials with positive and negative outcomes over selected time windows. **D.** There was no difference in voltage over midfrontal electrodes between trials with Go and NoGo responses. **E.** Over right occipital electrodes, the P3 was slightly stronger after Go than NoGo actions (no p-value because ROI selected based on visual inspection). **F.** Topoplots of difference in voltage between trials with Go and NoGo actions over selected time windows. ** $p < 0.01$. * $p < .05$, cluster-based permutation tests.

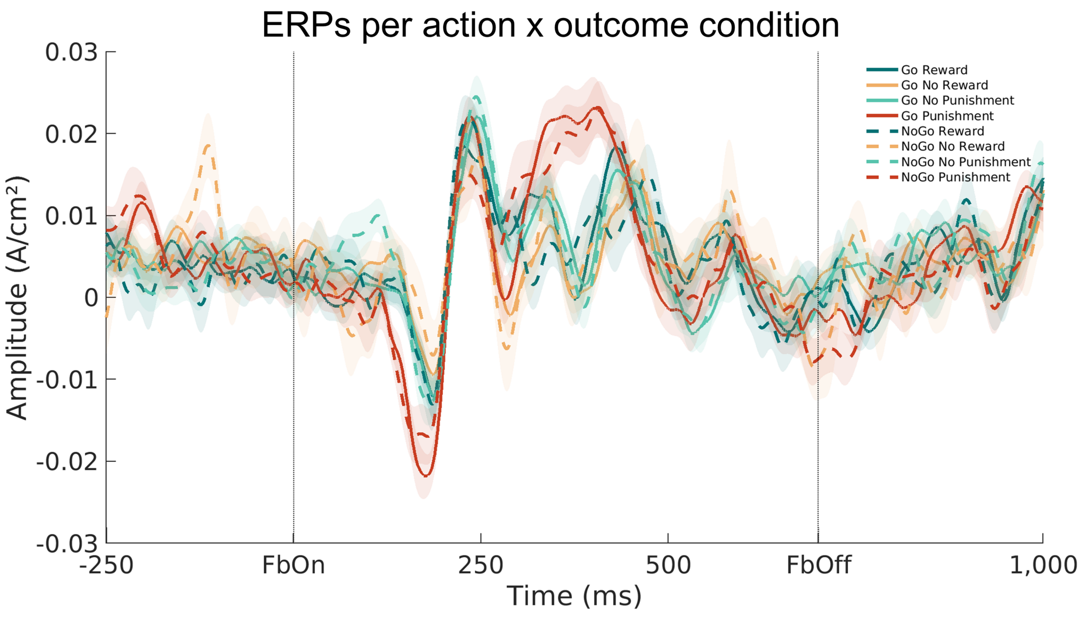

Supplementary Figure 15. ERPs per action x outcome condition. Error bands are ±SEM across participants, n=32. Biggest differences occurred around the time of the N2 (FRN) and P3 (RewP). N2 and P3 exhibited larger amplitudes on trials with punishments. There was no apparent modulation by the previous action (Go/ NoGo).

Supplementary Note 14: Model-based EEG analyses in the time domain

In addition to testing whether midfrontal time-frequency power reflected signatures of biased learning (see main text), we also tested whether the midfrontal time domain signal reflected biased learning. Again, we used the standard PE term and the difference term to biased PEs as regressors in a multiple linear regression on each channel-time bin.

Focusing on midfrontal electrodes, and controlling for outcomes valence, first, the PE_{STD} term was negatively correlated with midfrontal voltage around 529–575 ms (p = .039, two-tailed; Supplementary Fig. 14B). Note that so late after outcome onset, signal was not part of any “classical” ERP component any more. Second, the PE_{DIF} correlated negatively with midfrontal voltage around 123–166 ms (*p* = .029, two-tailed) in the time range of the N1 and later positively around 365–443 ms (*p* < .001, two-tailed; Supplementary Fig. 14C) in the time range of the P3/ RewP. Third, a similar pattern of correlations occurred for the PE_{BIAS} term (Cluster 1: negative, 111–184 ms, *p* = .004, two-tailed; Cluster 2: positive, 346–449 ms, *p* < .001, two-tailed; Supplementary Fig. 14A). Fourth, around these same time windows, midfrontal voltage also encoded outcome valence itself, but with opposite sign (Cluster 1: positive, 99–184 ms, *p* < .001, two-tailed; Cluster 2: negative, 308–448 ms, *p* < .001, two-tailed; see Supplementary Note 11 and Supplementary Fig. 12A).

In sum, similar to analyses of midfrontal power reported in the main text, PE sign and magnitude were encoded in midfrontal voltage around the same time, but with opposite polarity: Signal around the time of the N1 encoded PE sign positively, but PE magnitude negatively. Vice versa, signal around the time of the P3/ RewP encoded PE sign negatively, but PE magnitude positively. The same phenomenon of separate valence and magnitude encoding in midfrontal EEG signal has been reported before [12,13,19]. Notably, magnitude encoding in midfrontal voltage emerged for the PE_{BIAS} term, but not the PE_{STD}, indicating that this correlation was driven by the PE_{DIF} term and that biased learning described midfrontal voltage better than standard learning. These results complement our findings of theta/delta power encoding outcome valence and magnitude with opposite polarities (see main text).

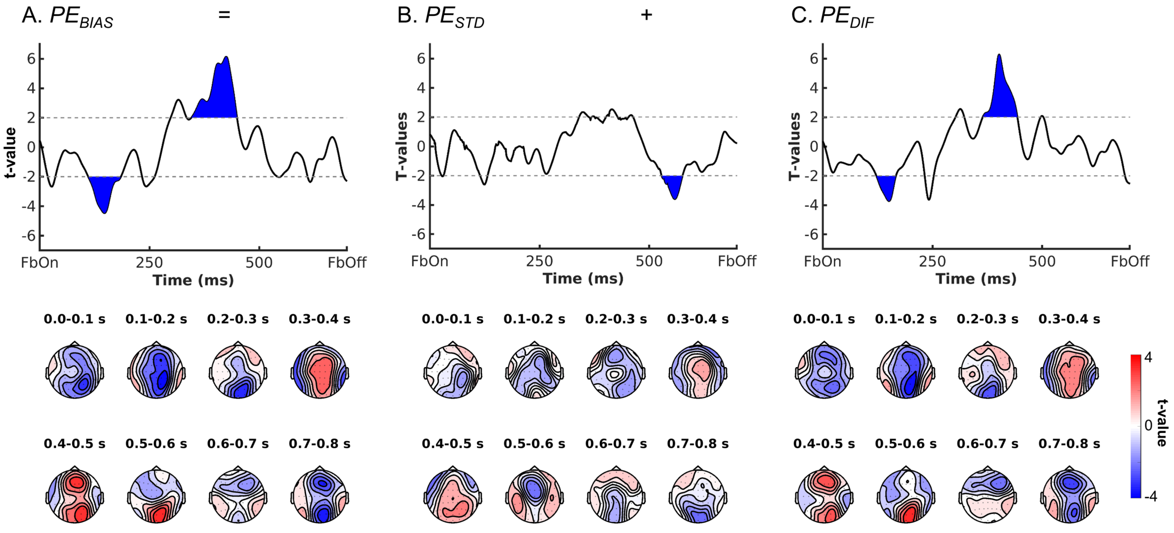

Supplementary Figure 16. Modulation of EEG voltage by biased PEs and decomposition into the standard PE term and the difference term to biased PEs. **A.** Mean EEG voltage over midfrontal electrodes (Fz, FCz, Cz) was significantly modulated by biased PEs around 111–184 (negatively; *p* < .001, two-tailed) and 353–414 ms (positively; *p* < .001, two-tailed) after outcome onset. **B.** Correlations with the standard PE term only emerged around 529–575 ms (negatively; *p* = .039, two-tailed). **C.** Correlations with the difference term to biased PEs were similar to correlations for the biased PE term itself, i.e., around 123–166 (negatively; *p* = .029, two-tailed) and 365–443 ms (positively; *p* < .001, two-tailed). Bottom row: Topoplots displaying *t*-values of beta-weights for the respective regressor over the entire scalp in steps of 100 ms from 0 to 800 ms.

Supplementary Note 15: Illustration of EEG-fMRI analysis fusion approaches

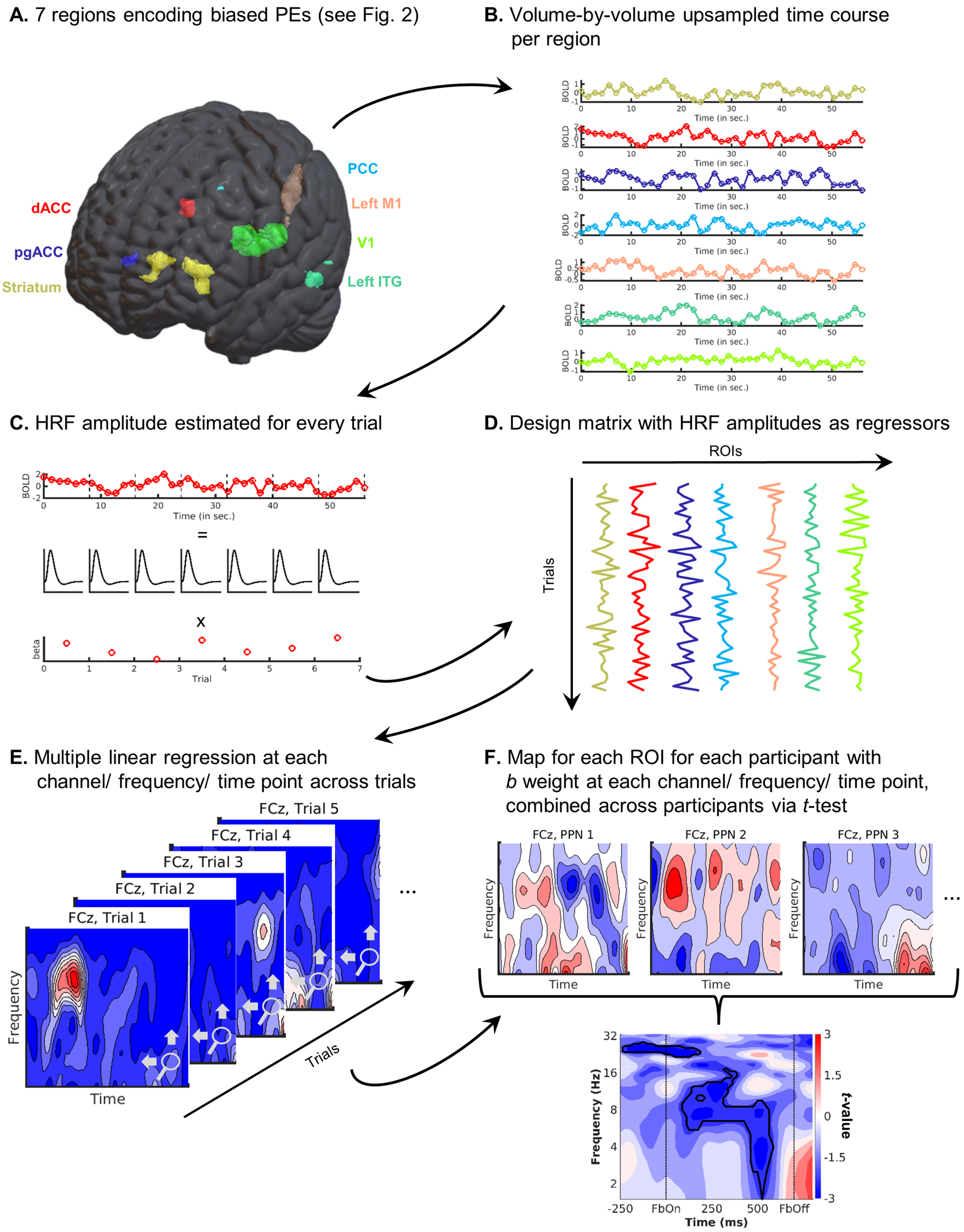

Supplementary Figure 17. Graphical illustration of the fMRI-informed EEG analysis approach. **A.** Regions are identified to encode biased PEs via a model-based GLM on BOLD data (see Fig. 2 in the main text). **B.** The volume-by-volume time-series of the signal in each ROI is extracted and upsampled. **C.** Time series are epoched into trials and the HRF amplitude is estimated for every trial. **D.** HRF amplitudes in every ROI for every trial are combined into a design matrix. **E.** The design matrix is applied in a multiple linear regression for each participant at each channel, frequency, and time point across trials. **F.** Regressions yield a sensor-frequency-time map of *b* regression weights for each ROI for each participant. Maps are combined across participants using a one-sample *t*-test.

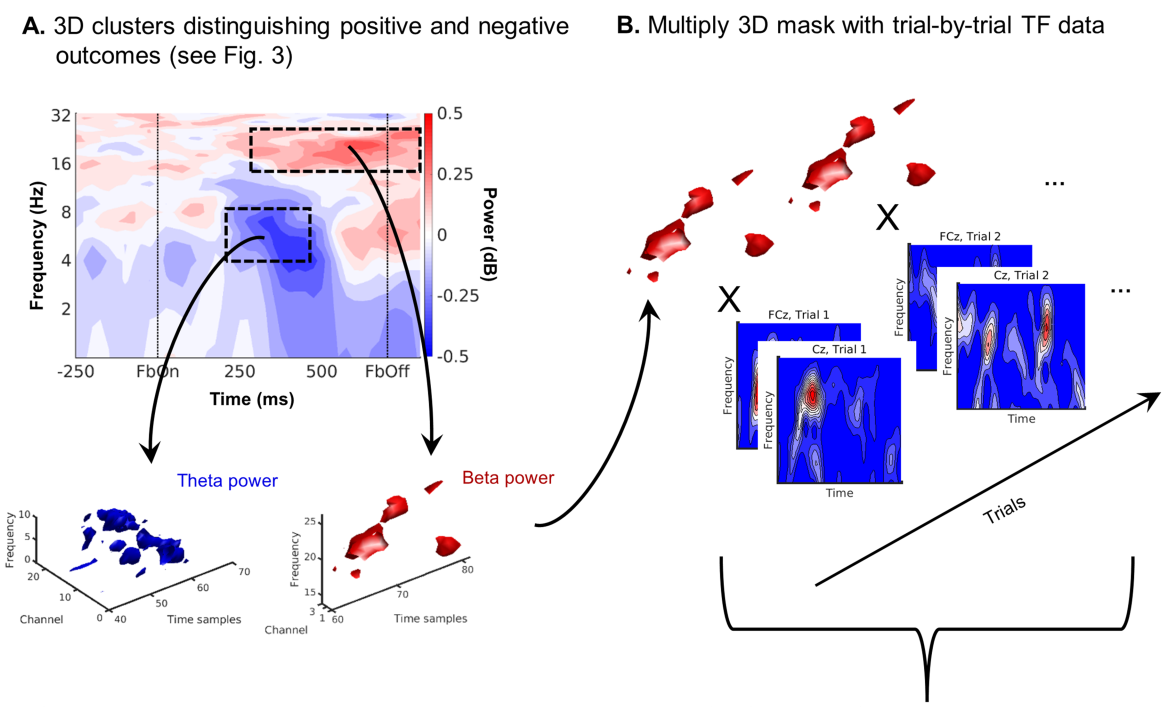

C. Trial-by-trial mean power in cluster added to design matrix for GLM on BOLD data

Trial	Action	Cue valence	Outcome	…	EEG
1	1	1	1	…	
2	0	0	-1	…	
3	0	1	0	…	
4	1	0	0	…	
5	1	1	1	…	
6	0	1	0	…	
7	1	0	-1	…	
8	1	0	-1	…	
9	0	1	1	…	
10	1	1	1	…	
….	…	…	…	…	…

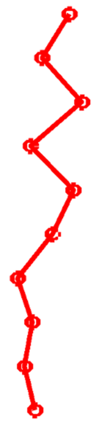

Supplementary Figure 18. Graphical illustration of the EEG-informed fMRI analysis approach. **A.** 3D clusters of channel-frequency-time points where power significantly distinguishes trials with positive from trials with negative outcomes are identified via a cluster-based permutation test (see Fig. 3A in the main text). The *t*-values above a threshold |2| are retained, weights at all other grid points are set to zero. **B.** The 3D t-value cluster is multiplied with the trial-by-trial channel-frequency-time data, yielding a single average value of power in the cluster at each trial. **C.** Trial-by-trial average power in the cluster is added as a parametric regressor in the GLM on BOLD-data and fitted with FSL.

Supplementary Note 16: fMRI-informed EEG results in time-frequency space

Besides the results for striatum, ACC, and PCC reported in the main text, there were also significant EEG correlates over midfrontal electrodes for trial-by-trial BOLD signal from left motor cortex (p = .002, two-tailed, around 0–625 ms, 16–27 Hz; Supplementary Fig. 17A). There were however no significant EEG correlates over midfrontal electrodes for BOLD signal from pgACC (p = .174, two-tailed; Fig. Supplementary Fig. 17B), left inferior temporal gyrus (p = .097, two-tailed; Supplementary Fig. 17C), and primary visual cortex (p = .170, two-tailed; Supplementary Fig. 17D).

As quality checks, we checked whether visual cortex BOLD correlated negatively with alpha over occipital electrodes [24,25] and whether motor cortex BOLD correlated negatively with beta power over central electrodes [26,27]. Both was the case (see Supplementary Fig. 17E, F), showing that our data was of sufficient quality to detect these well-established associations.

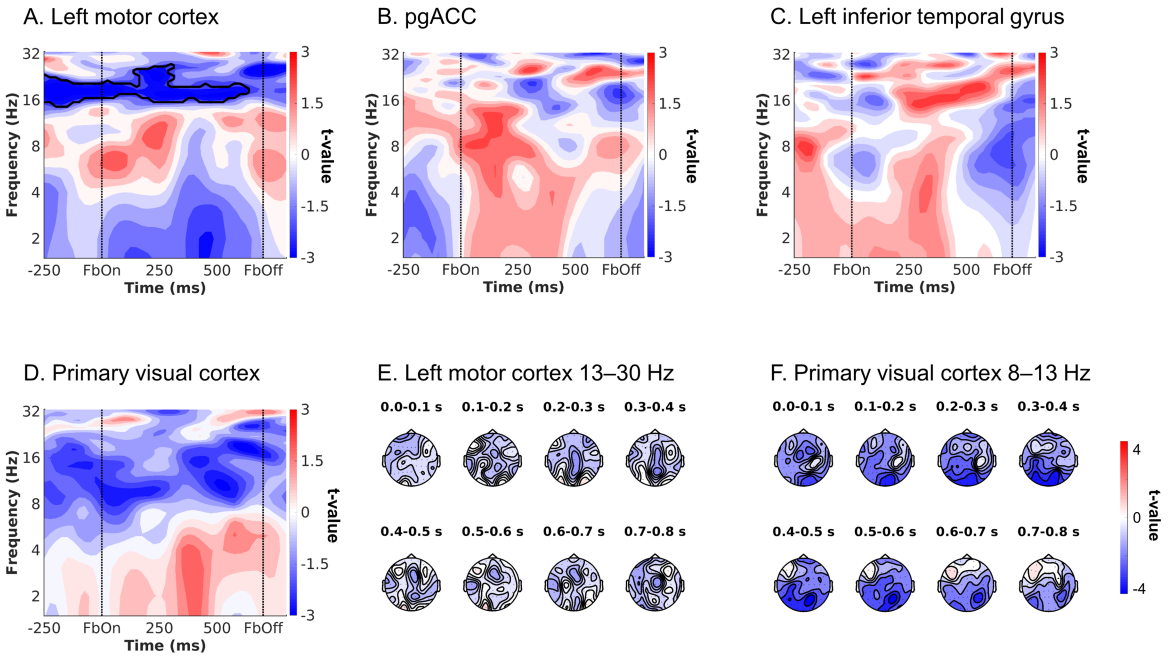

Supplementary Figure 19. Supplementary fMRI-informed EEG results in the time-frequency domain. Unique temporal contributions of BOLD signal in (**A**) left motor cortex, (**B**) pgACC, (**C**) left ITG and (**D**) primary visual cortex to midfrontal EEG power. Group-level t-maps display the modulation of the EEG power over midfrontal electrodes (Fz/ FCz/ Cz) by trial-by-trial BOLD signal in the selected ROIs. There significant correlations between midfrontal EEG TF power in the beta range and left motor cortex BOLD signal (p = .002; two-tailed), but no significant midfrontal EEG correlates for BOLD signal from other ROIs. **E.** Topoplots displaying t-values of left motor cortex BOLD over the entire scalp between 13 and 30 Hz (beta band) in steps of 100 ms from 0 to 800 ms. There were significant negatively correlates over central electrodes, especially round 300–500 ms. **F.** Topoplot displaying t-values of primary visual cortex BOLD over the entire scalp between 8 and 13 Hz (alpha band) in steps of 100 ms from 0 to 800 ms. There were significantly negatively correlations over occipital electrodes throughout outcome presentation.

Supplementary Note 17: fMRI-informed EEG results in the time domain

For fMRI-inspired analysis of the EEG signal in the time domain (voltage), we applied the same approach as reported in main text, but with voltage signal (time-domain) instead of time-frequency power as dependent variable. As independent variables, we entered the trial-by-trial BOLD signal from all seven regions encoding biased PEs plus the trial-by-trial standard PE and the different term towards the biased PE (exact same procedure as for EEG TF analyses), all in one single multiple linear regression. On a group-level, we again focused on the mean signal over midfrontal electrodes (Fz/ FCz/ Cz) in a time range of 0–700 ms, for which ERPs had been visible in the condition-averaged plots (see Supplementary Note 11 and Supplementary Fig. 12 and 13).

First, trial-by-trial striatal BOLD correlated significantly with midfrontal voltage at two time points, namely positively around 152–196 ms (p = .017, two-tailed) in the time range of the N1 and again negatively around 316–383 ms (p < .001, two-tailed, Supplementary Fig. 18A) in the time range of the N2/ FRN and P3/RewP. Second, trial-by-trial pgACC BOLD correlated significantly positively with midfrontal voltage around 347–412 ms (p = .006, two-tailed, Supplementary Fig. 18A) in the time range of the N2/ FRN and P3/RewP. Third, trial-by-trial BOLD from primary visual cortex correlated significantly positively with midfrontal voltage around 307–367 ms (p = .011, two-tailed, Supplementary Fig. 18B), overlapping with (but slightly earlier than) correlations from pgACC BOLD, i.e., in the time range of the N2/ FRN and P3/RewP. For midfrontal voltage split up per high vs. low BOLD signal (revealing which ERP components were respectively modulated), see Supplementary Fig. 18C-E. There were no significantly correlations between midfrontal voltage and trial-by-trial BOLD from dACC (p = .927, two-tailed, Supplementary Fig. 18A), left motor cortex (p = .649, two-tailed, Supplementary Fig. 18B), PCC (p = .796, two-tailed, Supplementary Fig. 18A), or left inferior temporal gyrus (p = .649, two-tailed, Supplementary Fig. 18B). For further details on BOLD-EEG voltage correlations in the time domain, see Supplementary Fig. 18F–L.

Taken together, trial-by-trial BOLD signal in striatum, pgACC, and V1 all correlated with FRN/ RewP amplitude, which was the dominant phenomenon over midfrontal electrodes reflecting outcome valence (see Supplementary Note 11 and Supplementary Fig. 12, 13). Notably, correlations with striatal and pgACC BOLD were of opposite signs, which aligns with the finding that striatal and pgACC BOLD predicted opposite behavioral tendencies on future trials (see main text; see Supplementary Fig. 20). However, crucially, the time domain signal did not allow for a temporal dissociation of these different regions. Possibly, the midfrontal evoked signal (i.e., the part of the signal that was phase-locked to outcome onset) was so stereotyped that only the FRN/ RewP complex showed enough variation across trials to allow for substantial correlations with trial-by-trial BOLD signal. This finding demonstrates that the time-frequency domain signal (i.e., the part of the signal that is not necessarily phase-locked to outcome onset) might be more suited for dissociating the activity of different regions in time.

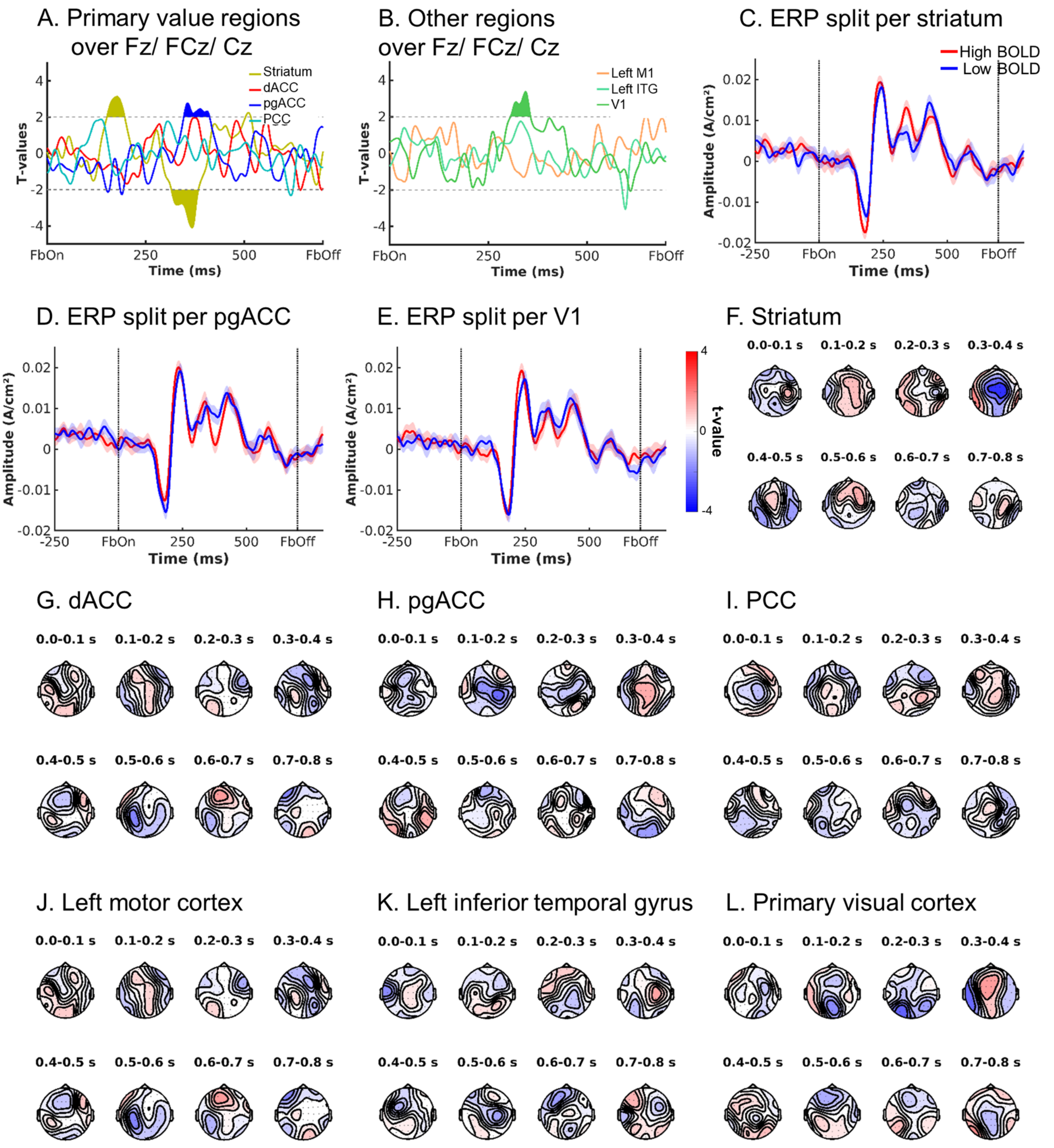

Supplementary Figure 20. fMRI-informed EEG analyses in the time-domain. Group-level *t*-value time courses display the modulation of the EEG voltage over midfrontal electrodes (Fz/ FCz/ Cz) by trial-by-trial BOLD signal in the selected ROIs. **A.** Correlations between midfrontal voltage and trial-by-trial BOLD signal from core value regions, i.e., striatum, dACC, pgACC, and PCC. Striatal BOLD modulates the amplitude of the N1 (152–196 ms; $p = .017$, two-tailed) and P3 (316–383 ms; $p < .001$, two-tailed), while the P3 amplitude was also modulated by pgACC BOLD (347–412 ms; $p = .006$, two-tailed). **B.** Correlations between midfrontal voltage and trial-by-trial BOLD signal from other regions, i.e., left motor cortex, left inferior temporal gyrus, and primary visual cortex. Visual cortex BOLD modulates the amplitude of the P3, as well. **C-E.** Midfrontal voltage split up for high vs. low BOLD signal (median split) from regions significantly modulating voltage. Striatal BOLD modulated N1 and P2 amplitude, while pgACC BOLD and visual cortex BOLD modulated N2 (FRN) amplitude. **F-L.** Topoplots displaying *t*-values of correlations between midfrontal voltage and trial-by-trial BOLD for all regions in steps of 100 ms from 0 to 800 ms.

Supplementary Note 18: Go/NoGo differences over time in BOLD signal, choices, alpha, and beta power

We observed differences between trials with Go responses and trials with NoGo responses in the low alpha power before and shortly after outcome onset (Fig. 6A, B main text). Alpha typically increases over the time course of an experiment, potentially related to fatigue and decreasing arousal [28]. If the ratio of Go and NoGo responses changed over time, as well, such an increase over time could spuriously lead to a difference between Go and NoGo responses (though note that this ratio did not noticeably change over time; Supplementary Fig. 19D). To exclude this possibility, we extracted trial-by-trial time-frequency power from the three significant clusters report in the main text in which power differed between Go and NoGo responses: i) lower alpha band power after outcome onset, ii) lower alpha band power before and after outcome onset, iii) beta band power before outcome onset. We log10-transformed this data to decibel and analyzed it as a function of the performed response (factor), block number (1–6; z-standardized), and the interaction between both. We reasoned that if power differences occurred merely due to fatigue effects, the main effect of performed response should not be significant when accounting for time on task (i.e., block number).

For lower alpha band power after outcome onset, there was a significant main effect of performed response, $\chi^2(1) = 5.350$, $p = .021$, $b = 0.035$, *95%-CI* [0.006, 0.064], two-tailed, with higher power for Go than NoGo responses, a significant main effect of block number with lower alpha band power increasing over time, $\chi^2(1) = 6.645$, $p = .010$, $b = 0.052$, *95%-CI* [0.015, 0.089], two-tailed, but no significant interaction, $\chi^2(1) = 0.156$, $p = .693$, $b = 0.003$, *95%-CI* [-0.013, 0.019], two-tailed. As Supplementary Fig. 19A reveals, lower alpha band power was consistently higher after Go than after NoGo responses for every block of the task, suggesting that differences in lower alpha band power were not merely due to time on task.

For lower alpha band power before and after outcome onset, as well, there was a significant main effect of performed response, $\chi^2(1) = 5.010$, $p = .025$, $b = 0.068$, *95%-CI* [0.009, 0.127], two-tailed, with higher power after Go than NoGo responses, a significant main effect of block number with lower alpha band power increasing over time, $\chi^2(1) = 6.757$, $p = .016$, $b = 0.072$, *95%-CI* [0.015, 0.129], two-tailed, but no significant interaction, $\chi^2(1) = 1.184$, $p = .277$, $b = 0.010$, *95%-CI* [-0.008, 0.028], two-tailed (Supplementary Fig. 19B), leading to identical conclusions.

For beta band power before and after outcome onset, there was a significant main effect of performed response, $\chi^2(1) = 6.301$, $p = .012$, $b = 0.083$, *95%-CI* [0.020, 0.146], two-tailed, with higher power after Go than NoGo responses, a significant main effect of block number with beta power decreasing over time, $\chi^2(1) = 4.007$, $p = .045$, $b = -0.042$, *95%-CI* [-0.083, -0.001], two-tailed, but no significant interaction, (1) = 0.030, $p = .864$, $b = 0.001$, *95%-CI* [-0.013, 0.015], two-tailed (Supplementary Fig. 19C). In sum, even in presence of changes in power over the time course of the task, lower alpha band and beta band power were consistently higher after Go responses than after NoGo responses, suggesting that these effects were not due to time on task.

Furthermore, we asked whether differences in dACC BOLD between trials with Go and trials with NoGo response at the time of the outcome were due to outcome-related activity or might rather the reflect action on the next trial. We thus plotted the "raw" BOLD signal per action x outcome condition. We used the first eigenvariate of the BOLD in signal in the dACC cluster that reflected biased learning, upsampled the BOLD signal, epoched it into trials relative to outcome onset (same procedure as for fMRI-informed EEG analyses), and averaged the signal across trials and participants separately per performed action (Go/NoGo) and outcome valence (positive/ negative). This plot yielded higher dACC BOLD signal on trials with NoGo responses than on trials with Go responses at the time of outcomes (Supplementary Fig. 19E). However, this difference could potentially be driven by the response on the

following task. Hence, we further split the data according to whether the action on the following trial was a Go or a NoGo response. Irrespective of the action on the following trial, dACC BOLD signal was higher when the action on the current trial was a NoGo response compared to a Go response (Supplementary Fig. 20F). In sum, these analyses corroborate that dACC BOLD signal was indeed higher after NoGo than Go responses at the time of outcomes.

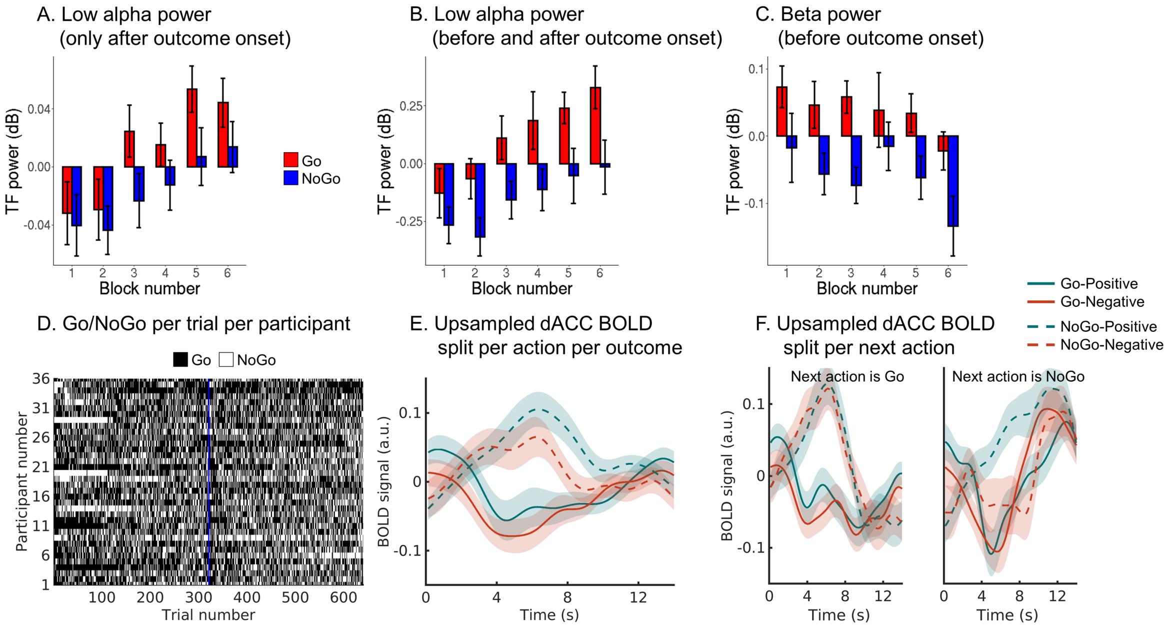

Supplementary Figure 21. Control analyses excluding temporal confounds in midfrontal lower alpha band power and dACC BOLD. **A.** Mean midfrontal low alpha power (±SEM across participants) after outcome onset, (**B**) before and after outcome onset, and (**C**) beta power before outcome onset as a function of the performed action and block number (i.e., time on task; error bars are ±SEM across participants, n=32). While low alpha power increases ($\chi^2(1) = 6.645$, $p = .010$, $b = 0.052$, *95%-CI* [0.015, 0.089], two-tailed) and beta power decreases ($\chi^2(1) = 4.007$, $p = .045$, $b = -0.042$, *95%-CI* [-0.083, -0.001], two-tailed) over the time course of the task, power was always consistently higher for trials with Go than trials with NoGo responses (alpha power: $\chi^2(1) = 5.350$, $p = .021$, $b = 0.035$, *95%-CI* [0.006, 0.064], two-tailed; beta power: $\chi^2(1) = 6.301$, $p = .012$, $b = 0.083$, *95%-CI* [0.020, 0.146], two-tailed), suggesting that action effects were not reducible to time on task. **D.** Response for each participant (rows) on each trial (columns). There was no noticeable change in the overall ratio of Go to NoGo responses over time. The vertical blue line indicates the start of the second session featuring new stimuli. **E.** Mean upsampled dACC BOLD signal (error bars are ±SEM across participants) at the time of the outcome, split per performed action (Go/NoGo) and outcome valence (positive/negative). BOLD signal was higher after NoGo than Go responses. **F.** Same plot as (E), but split based on whether the next action was a Go (left panel) or an NoGo (right panel) response. Even if the next response was NoGo, BOLD signal was higher for trials with NoGo responses (on the current trial) than trials Go responses.

Supplementary Note 19: Stay behavior as a function of BOLD and EEG TF power

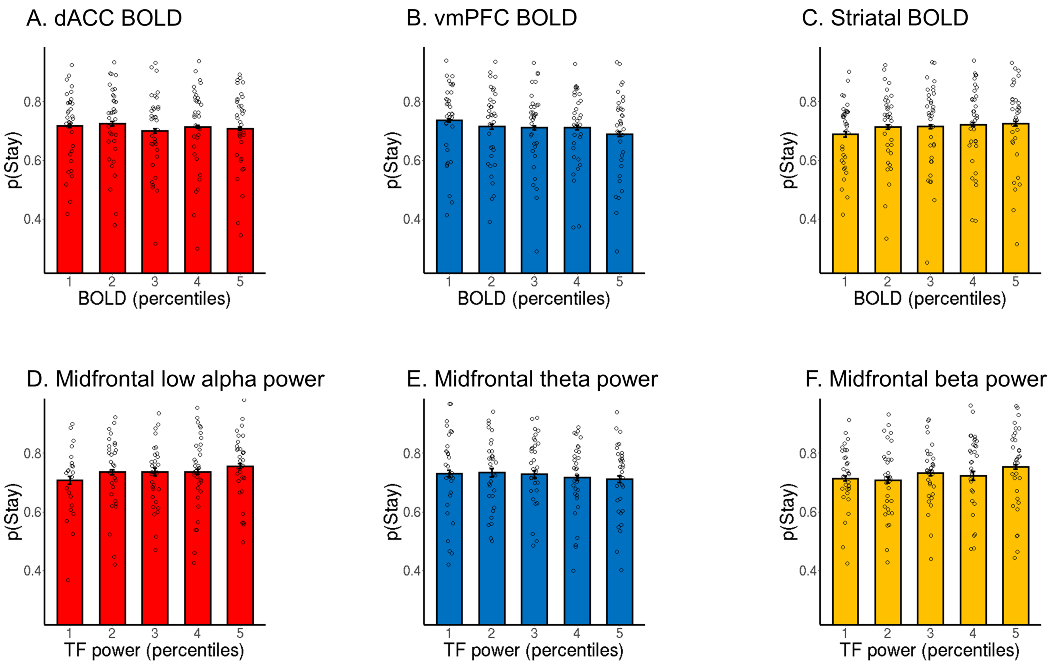

Supplementary Figure 22. Probability of repeating the same response ("stay") on the next cue encounter as a function of outcome-related BOLD and EEG signal. **A-C.** Probability of repeating the same action ("staying") as a function of BOLD signal from (**A**) dACC, (**B**) vmPFC (cluster correlating with theta power in Fig. 5F), and (**C**) striatum (split into 5 bins; error bands are ±SEM across participants, n=32). While dACC BOLD was not significantly linked to the probability to stay ($\chi^2(1) = 1.294$, $p = .255$, $b = -0.019$, *95%-CI* [-0.050, 0.012], two-tailed), high BOLD signal in vmPFC predicted a higher chance to switch to another action ($\chi^2(1) = 8.765$, $p = .003$, $b = -0.065$, *95%-CI* [-0.104, -0.026], two-tailed), while high BOLD signal in striatum predicted a higher probability of staying with the same action ($\chi^2(1) = 9.051$, $p = .003$, $b = 0.067$, *95%-CI* [0.020, 0.114], two-tailed). **D-E.** Probability of staying as a function of midfrontal time-frequency power in the (**D**) low alpha, (**E**) theta/delta, and (**F**) beta range. Higher low alpha power ($\chi^2(1) = 10.711$, $p = .001$, $b = 0.179$, *95%-CI* [0.077, 0.281], two-tailed) and higher beta power ($\chi^2(1) = 11.886$, $p < .001$, $b = 0.145$, *95%-CI* [0.065, 0.225], two-tailed) predict a higher probability of staying with the same action, while higher theta power predicts a higher chance to switch to another action ($\chi^2(1) = 4.179$, $p = .041$, $b = -0.099$, *95%-CI* [-0.191, -0.007], two-tailed). Grey circles represent individual per condition-per-participant means. Error bars were very narrow (and thus hardly visible) and computed based on the Cousineau-Morey methods based on per-condition-per-participant means.

Supplementary Table 1: Stay behavior as a function of action, salience, and valence

Effect	χ^2	Df	*p*-value	*b*	*95%-CI*
Action	0.01	1	.924	-0.005	-0.115, 0.105
Salience	5.15	1	.021	0.058	0.009, 0.107
Valence	45.59	1	< .001	-0.504	-0.608, -0.400
Action x Salience	0.12	1	.728	-0.011	-0.074, 0.052
Action x Valence	3.24	1	.067	-0.062	-0.129, 0.005
Salience x Valence	30.95	1	< .001	0.450	-0.575, -0.325
Action x Valence x Salience	19.73	1	< .001	0.248	0.154, 0.342
Salient outcomes only:					
Action	0.01	1	.960	0.004	-0.129, 0.137
Valence	46.36	1	< .001	-0.957	-1.151, -0.763
Action x Valence	17.80	1	< .001	-0.308	-0.433, -0.183
Neutral outcomes only:					
Action	.102	1	.750	-0.019	-0.137, 0.099
Valence	.830	1	.362	-0.056	-0.176, 0.064
Action x Valence	12.32	1	< .001	0.188	0.092, 0.284
Go with salient outcomes only:					
Valence	53.93	1	< .001	-1.276	-1.501, -1.051
NoGo with salient outcomes only:					
Valence	18.23	1	< .001	-0.637	-0.886, -0.388
Go with neutral outcomes only:					
Valence	0.13	1	.050	0.134	0.001, 0.267
NoGo with neutral outcomes only:					
Valence	7.21	1	.007	-0.244	-0.415, -0.073

Supplementary Table 1. Full report of model of stay behavior. Mixed-effects logistic regression of stay vs. switch behavior (i.e., repeating vs. changing an action on the next occurrence of the same cue) as a function of performed action (Go vs. NoGo), outcome salience (salient: reward or punishment vs. neutral: no reward or no punishment), and outcome valence (positive: reward or no punishment vs. negative: no reward or punishment). Follow-up analyses were performed on trials with salient vs. neutral outcomes separately, and then separately based on Go vs. NoGo actions and salient vs. neutral outcomes. *P*-values were computed using likelihood ratio tests using the *mixed*-function (option "LRT") from package *afex*. All tests were two-tailed. No adjustments were made for multiple comparisons given that distinct hypotheses were tested.

Supplementary Table 2: Model parameters and fit indices for models M1-M6

	M1	M2	M3	M4	M5 (Asymmetric pathways)	M6 (Action priming)
Mean log model evidence	-609.30	-597.95	-554.46	-532.40	-528.13	-540.84
Model frequency	0	0.0278	0	0.0488	0.6815	0.2419
Protected exceedance probability	0	0	0	0	.9970	.0030
ρ	7.75 [0.53 – 38.68]	6.81 [0.48 – 37.74]	6.38 [0.49 – 35.71]	10.05 [1.26 – 40.60]	9.41 [0.98 – 31.22]	6.64 [0.71 – 22.83]
ε_0	0.17 [0.002 – 0.77]	0.20 [0.003 – 0.82]	0.21 [0.003 – 0.85]	0.09 [0.003 – 0.38]	0.08 [0.003 – 0.41]	0.039 [0.003 – 0.11]
b		-0.05 [-1.23 – 0.82]	-0.01 [-1.23 – 1.09]	0.13 [-1.16 – 1.03]	0.14 [-1.18 – 1.10]	0.16 [-1.22 – 1.40]
π			0.77 [-0.78 – 3.73]		0.17 [-1.25 – 2.70]	-1.11 [-3.29 – 1.23]
$\varepsilon_{\text{rewarded Go}}$ ($\varepsilon_0+\kappa$)				0.749 [0.29 – 0.99]	0.833 [0.43 – 0.99]	
$\varepsilon_{\text{punished NoGo}}$ ($\varepsilon_0-\kappa$)				0.001 [0.001 – 0.02]	0.003 [0.001 – 0.09]	
$\varepsilon_{\text{salient Go}}$						0.49 [0.05 – 0.90]

Supplementary Table 2. Model parameters for fitted models. Mean [minimum – maximum] of participant-level parameter estimates in model space, fitted with hierarchical Bayesian inference (only the respective model included in the fitting process). Model frequency and protected exceedance probability were based on a model comparison that involves models M1-M6. Note that Fig. 2 in the main text does not include M6.

Supplementary Table 3: BOLD-GLM with parametric modulation by standard and biased prediction errors

	Regressor	1	2	3	4	5	6	7	8	9	10
	Contrast	WinGoOnset	AvoidGoOnset	WinNoGoOnset	AvoidNoGoOnset	Handedness	Error	Outcome Onset	PE_{STD}	PE_{DIF}	Invalid
1	PE_{STD}								1		
2	PE_{DIF}									1	

Supplementary Table 3. BOLD-GLM with parametric modulation by standard and biased prediction errors. Explanation of regressors:

WinGoOnset: for every trial with Win cue and Go action, at cue onset, duration 1, value +1.

AvoidGoOnset: for every trial with Avoid cue and Go action, at cue onset, duration 1, value +1.

WinNoGoOnset: for every trial with Win cue and NoGo action, at cue onset, duration 1, value +1.

AvoidNoGoOnset: for every trial with Avoid cue and NoGo action, at cue onset, duration 1, value +1.

Handedness: for every trial, at cue onset, duration 1, value +1 for left hand response, 0 for NoGo 10 response, -1 for right hand response.

Error: for every trial, at cue onset, duration 1, value +1 for incorrect response, 0 for correct response.

OutcomeOnset: for every trial, at outcome onset, duration 1, value +1 for every trial.

PE_{STD}: for every trial, at outcome onset, duration 1, value is the demeaned PE times learning rate for model M1.

PE_{DIF}: for every trial, at outcome onset, duration 1, value is the demeaned difference between (PE times learning rate) for model M1 and (PE times learning rate) for model M5.

Invalid: for trials where uninstructed button was pressed, at outcome onset, duration 1, value 1.

Supplementary Table 4: BOLD-GLM with response-locked and outcome-locked categorical regressors

	Regressors	1	2	3	4	5	6	7	8	9	10	11	12	13
	Contrast	GoReward	GoNoReward	GoNoPunishment	GoPunishment	NoGoReward	NoGoNoReward	NoGoNoPunishment	NoGoPunishment	LeftHand	RightHand	Error	OutcomeOnset	Invalid
1	Valence	1	-1	1	-1	1	-1	1	-1					
2	Action	1	1	1	1	-1	-1	-1	-1					
3	Hand Sum									1	1			
4	Hand Dif									1	-1			

Supplementary Table 4. BOLD-GLM with response-locked and outcome-locked categorical regressors. Explanation of regressors:

GoReward: for every trial with Go action and reward obtained, at outcome onset, duration 1, value +1.
GoNoReward: for every trial with Go action and no reward obtained, at outcome onset, duration 1, value +1.
GoNoPunishment: for every trial with Go action and no punishment obtained, at outcome onset, duration 1, value +1.
GoPunishment: for every trial with Go action and punishment obtained, at outcome onset, duration 1, value +1.
NoGoReward: for every trial with NoGo action and reward obtained, at outcome onset, duration 1, value +1.
NoGoNoReward: for every trial with NoGo action and no reward obtained, at outcome onset, duration 1, value +1.
NoGoNoPunishment: for every trial with NoGo action and no punishment obtained, at outcome onset, duration 1, value +1.
NoGoPunishment: for every trial with NoGo action and punishment obtained, at outcome onset, duration 1, value +1.
LeftHand: for very trial with left hand response, at response onset, duration 1, value + 1.
RightHand: for very trial with right hand response, at response onset, duration 1, value + 1.
Error: for every trial, at cue onset, duration 1, value +1 for incorrect response, 0 for correct response.
OutcomeOnset: for every trial, at outcome onset, duration 1, value +1 for every trial.
Invalid: for trials where uninstructed button was pressed, at outcome onset, duration 1, value 1.

Supplementary Table 5: Significant clusters in BOLD-GLM with parametric modulation by standard and biased prediction errors

No	Contrast<br>Brain region	Maximal Z-value	Cluster size (voxels)	Corrected p	Peak coordinates<br>x	y	z
	**PE_{STD} Positive**						
1	Ventromedial prefrontal cortex, Nucleus accumbens, caudate, putamen, bilateral amygdala, bilateral hippocampus	6.47	8762	1.02e-43	12	14	-6
2	Occipital pole, lingual gyrus, occipital fusiform gyrus	6.64	1012	6.10e-10	10	-92	-10
3	Posterior cingulate cortex	4.72	985	9.40e-10	4	-50	18
4	Left superior frontal gyrus	5.56	910	3.19e-09	-18	34	50
5	Right middle temporal gyrus, anterior division	5.48	381	6.47e-05	62	-4	-18
6	Left inferior temporal gyrus, temporooccipital part	5.16	360	.000103	-52	-46	-10
7	Left middle temporal gyrus, anterior division	4.70	329	.000209	-60	-10	-14
8	Left postcentral gyrus	4.33	271	.000838	-52	-28	48
9	Right cerebellum	4.89	147	.0239	44	-72	-40
10	Anterior cingulate cortex	4.27	146	.0247	2	6	34
	**PE_{STD} Negative**						
1	Right superior frontal gyrus	5.20	351	.000127	6	26	62
2	Right occipital pole, right inferior lateral occipital cortex	4.76	211	.00391	30	-94	4
3	Left lingual gyrus	4.21	186	.00776	-22	-64	2
4	Left inferior lateral occipital cortex	4.28	147	.0239	-44	-86	-10
	**PE_{DIF} Positive**						
1	Bilateral superior frontal gyrus, paracingulate gyrus, anterior cingulate cortex, posterior cingulate cortex, ventromedial frontal cortex, bilateral frontal orbital cortex, bilateral frontal pole, bilateral supramarginal gyrus, bilateral middle temporal gyrus, bilateral inferior temporal gyrus, bilateral fusiform gyrus, bilateral inferior occipital cortex, bilateral superior occipital cortex, precuneous, bilateral cerebellum	7.11	35109	0	34	-84	20
2	Right insula, right frontal operculum, right inferior frontal gyrus, right middle frontal gyrus, right frontal orbital cortex, bilateral caudate, bilateral Nucleus accumbens, bilateral thalamus, brainstem	6.36	10364	0	34	20	-8
3	Left insula, left frontal operculum, left inferior frontal gyrus, left middle frontal gyrus, left frontal orbital cortex	6.51	10132	0	-36	20	-6

4	Right middle temporal gyrus, posterior division	4.66	307	.0003	56	-32	-4
5	Right insula, right planum polare	4.72	143	.0248	40	-8	-12
	**PE_{DIF} Negative**						
1	Left middle temporal gyrus, anterior division	4.22	191	.00607	-64	-6	-14
2	Left hippocampus	4.49	158	.0158	-26	-14	-22

Significant clusters in BOLD-GLM with parametric modulation by standard and biased prediction errors. *P*-values reflect cluster-based tests (testing clusters of coefficients above the cluster-forming threshold of $|z| > 3.1$ against a reference null distribution). Two separate one-tailed tests (testing separately for negative and positive clusters) at $\alpha = .025$ were performed.

Supplementary Table 6: Significant clusters in BOLD-GLM with response-locked and outcome-locked categorical regressors

	Contrast				Peak coordinates		
No	Brain region	Maximal Z-value	Cluster size (voxels)	Corrected p	x	y	z
	**Positive > Negative**						
1	Ventromedial prefrontal cortex, left lateral orbitofrontal cortex, Nucleus accumbens, caudate, putamen, bilateral amygdala, bilateral hippocampus	5.65	3999	2.86e-19	8	12	-4
2	Left superior frontal gyrus	4.03	331	0.00239	-18	28	60
3	Left lateral orbitofrontal cortex	4.31	288	0.00512	-34	40	-8
4	Right occipital pole	4.59	213	0.0212	18	-92	-16
	**Negative > Positive**						
1	Right lateral orbitofrontal cortex	4.59	367	0.00142	30	62	-2
2	Precuneous	4.58	356	0.00170	8	-66	58
3	Right superior frontal gyrus	4.32	340	0.00223	12	14	72
	**Go > NoGo outcome-locked**						
	No significant clusters						
	**NoGo > Go outcome-locked**						
1	Bilateral lateral orbitofrontal cortex, Bilateral superior frontal gyrus, anterior cingulate cortex, posterior cingulate cortex, pre-SMA, bilateral precentral gyrus, bilateral postcentral gyus, bilateral supramarginal gyrus, bilateral operculum, bilateral planum temporale, bilateral superior temporal gyrus, bilateral middle temporal gyrus, bilateral inferior temporal gyrus, bilateral superior lateral occipital cortex, bilateral inferior lateral occipital cortex, bilateral thalamus	7.32	114090	0	-42	-6	12
	**Go (left + right hand response) > NoGo response-locked**						
1	Cerebellum, bilateral thalamus, bilateral putamen, bilateral caudate, bilateral Nucleus Accumbens, posterior cingulate cortex, right operculum, right angular gyrus, right superior parietal lobule. anterior cingulate cortex, paracingulate gyrus, bilateral ventrolateral frontal cortex, right middle frontal gyrus	7.08	46437	0	32	-4	-6
2	Left operculum, left angular gyrus, left superior parietal lobule	5.88	3936	3.13e-17	-46	-24	26
3	Intracalcarine cortex	3.79	374	0.00248	-12	-88	6
4	Right middle temporal gyrus	4.63	287	0.00956	68	-32	-12

	**NoGo > Go (left + right hand response) response-locked**						
1	Right medial temporal gyrus, right temporal pole	4.09	465	0.000636	50	-8	-16
2	vmPFC, subcallosal cortex	3.95	435	0.000973	0	40	-12
	**Left Hand > Right Hand Response response-locked**						
1	Right precentral gyrus, right postcentral gyrus, right superior parietal lobule, right operculum	7.05	9460	9.41e-39	46	-24	64
2	Left cerebellum	7.18	2208	2.1e-14	-18	-54	-18
	**Right Hand > Left Hand Response response-locked**						
1	left precentral gyrus, left postcentral gyrus, left superior parietal lobule, left operculum, left thalamus	7.06	14870	0	-36	-20	66
2	Right anterior cerebellum	7.90	3735	1.44e-20	18	-54	-20
3	Right inferior lateral occipital cortex, right superior lateral occipital cortex	4.96	1452	9.66e-11	48	-86	-4
4	Right angular gyrus	4.98	551	2.06e05	66	-50	28
5	Left occipital pole, right intracalcarine cortex	3.93	409	0.000236	-4	-96	26
6	Right posterior cerebellum	4.64	200	0.0157	48	-78	-32

Significant clusters in BOLD-GLM with response-locked and outcome-locked categorical regressors. *P*-values reflect cluster-based tests (testing clusters of coefficients above the cluster-forming threshold of $|z| > 3.1$ against a reference null distribution). Two separate one-tailed tests (testing separately for negative and positive clusters) at $\alpha = .025$ were performed.

Supplementary Table 7: Significant clusters in BOLD-GLM with EEG regressors

No	Contrast<br>Brain region	Maximal Z-value	Cluster size (voxels)	Corrected p	Peak coordinates<br>x	 y	 z
	**Central Lower Alpha Band Positive**<br>*No significant clusters*						
	**Central Lower Alpha Band Negative**						
1	Precuneous,<br>cuneal cortex,<br>right superior lateral occipital cortex	5.78	8346	2.50e-33	6	-60	66
2	Anterior cingulate gyrus,<br>right superior frontal gyrus	4.77	2449	1.75e-14	24	12	66
3	Left middle frontal gyrus,	5.59	1828	7.63e-12	-38	8	34
4	Right insula,<br>right central opercular cortex	4.71	1794	1.08e-11	42	2	28
5	Right frontal pole,<br>right middle frontal gyrus,<br>right inferior frontal gyrus, pars triangularis	5.43	1300	2.37e-09	30	40	20
6	Left supramarginal gyrus, anterior division	4.61	959	1.19e-07	-64	-36	42
7	Left angular gyrus	5.83	916	2.38e-07	-48	-52	18
8	Right cerebellum, anterior	4.79	480	.000131	42	-38	-38
9	Posterior cingulate cortex,<br>parahippocampal gyrus,<br>right thalamus	4.41	424	.000328	14	-38	-2
10	Left temporal pole,<br>left inferior frontal gyrus, pars opercularis<br>left insula	4.08	413	.000394	-56	16	-6
11	Left cerebellum, anterior	5.44	263	.00598	-30	-40	-42
12	Right lingual gyrus	3.43	235	.0104	10	-74	-10
13	Left cerebellum, posterior	5.74	215	.0158	-14	-76	-42
14	Brainstem	4.35	207	.0186	8	-34	-20
	**Frontal Theta Band Positive**						
1	Right bilateral precentral gyrus	4.82	394	.000577	12	-16	80
2	Left bilateral precentral gyrus	5.25	357	.0011	-20	-28	78
	**Frontal Theta Band Negative**						
1	Right supramarginal gyrus, posterior division,<br>right superior lateral occipital cortex	3.94	1002	1.10e-07	-54	-50	44
2	Left supramarginal gyrus, posterior division,<br>Left superior lateral occipital cortex	4.39	508	8.96e-05	56	-50	20
3	Posterior cingulate cortex	4.58	419	.000378	-6	-30	38
4	Ventromedial prefrontal cortex	4.03	342	.00143	0	42	4
	**Central Beta Band Positive**						
1	Right caudate	4.19	258	.00481	16	30	6
2	Left parahippocampal gyrus, posterior divison	4.86	221	.0106	-38	-36	-8
	**Central Beta Band Negative**						
1	Right frontal pole,<br>right middle frontal gyrus,<br>right superior frontal gyrus	5.49	6599	7.06e-30	-32	8	28
2	Left frontal pole,<br>left middle frontal gyrus,<br>Left superior frontal gyrus	5.51	6144	1.82e-28	40	38	36
3	Left supramarginal gyrus, posterior division,<br>left superior parietal lobule,<br>left superior lateral occipital cortex,<br>Left middle temporal gyrus, temporooccipital part	5.51	5175	2.43e-25	-66	-44	28

4	Right supramarginal gyrus, posterior division, Right superior parietal lobule, right superior lateral occipital cortex	5.13	3264	1.62e-18	30	-74	54
5	Left superior frontal gyrus, paracingulate gyrus, precuneous	4.54	1235	1.80e-09	-4	12	52
6	Right superior temporal gyrus, posterior division	4.59	1076	1.33e-08	48	-14	-10
7	Left temporal pole, left planum temporale	4.96	320	.00139	-46	4	-18

Significant clusters in BOLD-GLM with EEG regressors. P-values reflect cluster-based tests (testing clusters of coefficients above the cluster-forming threshold of $|z| > 3.1$ against a reference null distribution). Two separate one-tailed tests (testing separately for negative and positive clusters) at $\alpha = .025$ were performed.

Supplementary References

1. Wilson, R. C. & Niv, Y. Is model fitting necessary for model-based fMRI? *PLOS Computational Biology* **11**, e1004237 (2015).
2. Palminteri, S., Wyart, V. & Koechlin, E. The importance of falsification in computational cognitive modeling. *Trends in Cognitive Sciences* **21**, 425–433 (2017).
3. Nassar, M. R. & Frank, M. J. Taming the beast: Extracting generalizable knowledge from computational models of cognition. *Current Opinion in Behavioral Sciences* **11**, 49–54 (2016).
4. Wilson, R. C. & Collins, A. G. Ten simple rules for the computational modeling of behavioral data. *eLife* **8**, 1–35 (2019).
5. Frank, M. J. Dynamic dopamine modulation in the basal ganglia: A neurocomputational account of cognitive deficits in medicated and nonmedicated Parkinsonism. *Journal of Cognitive Neuroscience* **17**, 51–72 (2005).
6. Collins, A. G. E. & Frank, M. J. Opponent actor learning (OpAL): Modeling interactive effects of striatal dopamine on reinforcement learning and choice incentive. *Psychological Review* **121**, 337–366 (2014).
7. Cockburn, J., Collins, A. G. E. & Frank, M. J. A reinforcement learning mechanism responsible for the valuation of free choice. *Neuron* **83**, 551–557 (2014).
8. Rutledge, R. B. *et al.* Dopaminergic drugs modulate learning rates and perseveration in Parkinson's patients in a dynamic foraging task. *Journal of Neuroscience* **29**, 15104–15114 (2009).
9. Behrens, T. E. J., Woolrich, M. W., Walton, M. E. & Rushworth, M. F. S. Learning the value of information in an uncertain world. *Nature Neuroscience* **10**, 1214–1221 (2007).
10. Cohen, M. X. & Donner, T. H. Midfrontal conflict-related theta-band power reflects neural oscillations that predict behavior. *Journal of Neurophysiology* **110**, 2752–2763 (2013).
11. Cohen, M. X., Wilmes, K. A. & van de Vijver, I. Cortical electrophysiological network dynamics of feedback learning. *Trends in Cognitive Sciences* **15**, 558–566 (2011).
12. Bernat, E. M., Nelson, L. D. & Baskin-Sommers, A. R. Time-frequency theta and delta measures index separable components of feedback processing in a gambling task. *Psychophysiology* **52**, 626–637 (2015).
13. Cavanagh, J. F. Cortical delta activity reflects reward prediction error and related behavioral adjustments, but at different times. *NeuroImage* **110**, 205–216 (2015).
14. Proudfit, G. H. The reward positivity: From basic research on reward to a biomarker for depression. *Psychophysiology* **52**, 449–459 (2015).
15. Paul, K., Vassena, E., Severo, M. C. & Pourtois, G. Dissociable effects of reward magnitude on fronto-medial theta and FRN during performance monitoring. *Psychophysiology* **57**, e13481 (2020).
16. Sambrook, T. D. & Goslin, J. Principal components analysis of reward prediction errors in a reinforcement learning task. *NeuroImage* **124**, 276–286 (2016).
17. Yeung, N. & Sanfey, A. G. Independent coding of reward magnitude and valence in the human brain. *Journal of Neuroscience* **24**, 6258–6264 (2004).
18. Kreussel, L. *et al.* The influence of the magnitude, probability, and valence of potential wins and losses on the amplitude of the feedback negativity. *Psychophysiology* **49**, 207–219 (2012).
19. Talmi, D., Atkinson, R. & El-Deredy, W. The feedback-related negativity signals salience prediction errors, not reward prediction errors. *Journal of Neuroscience* **33**, 8264–8269 (2013).
20. Sato, A. *et al.* Effects of value and reward magnitude on feedback negativity and P300. *NeuroReport* **16**, 407–411 (2005).
21. Tanner, D., Morgan-Short, K. & Luck, S. J. How inappropriate high-pass filters can produce artifactual effects and incorrect conclusions in ERP studies of language and cognition. *Psychophysiology* **52**, 997–1009 (2015).
22. Foti, D., Weinberg, A., Dien, J. & Hajcak, G. Event-related potential activity in the basal ganglia differentiates rewards from nonrewards: Temporospatial principal components analysis and source localization of the feedback negativity. *Human Brain Mapping* **32**, 2207–2216 (2011).

23. Wu, Y. & Zhou, X. The P300 and reward valence, magnitude, and expectancy in outcome evaluation. *Brain Research* **1286**, 114–122 (2009).
24. Scheeringa, R. *et al.* Neuronal dynamics underlying high-and low-frequency EEG oscillations contribute independently to the human BOLD signal. *Neuron* **69**, 572–583 (2011).
25. Zumer, J. M., Scheeringa, R., Schoffelen, J.-M., Norris, D. G. & Jensen, O. Occipital alpha activity during stimulus processing gates the information flow to object-selective cortex. *PLoS Biology* **12**, e1001965 (2014).
26. Jurkiewicz, M. T., Gaetz, W. C., Bostan, A. C. & Cheyne, D. Post-movement beta rebound is generated in motor cortex: Evidence from neuromagnetic recordings. *NeuroImage* **32**, 1281–1289 (2006).
27. Ritter, P., Moosmann, M. & Villringer, A. Rolandic alpha and beta EEG rhythms' strengths are inversely related to fMRI-BOLD signal in primary somatosensory and motor cortex. *Human Brain Mapping* **30**, 1168–1187 (2009).
28. Klimesch, W. EEG alpha and theta oscillations reflect cognitive and memory performance: A review and analysis. *Brain Research Reviews* **29**, 169–195 (1999).
