# Supplementary material for: Prefrontal signals precede striatal signals for biased credit assignment in motivational learning biases
Source: Nat Commun. 2024 Jan 2;15:19. doi: 10.1038/s41467-023-44632-x (PMC10762147; doi:10.1038/s41467-023-44632-x)
Supplement: Supplementary file 3 — Reporting Summary [file 41467_2023_44632_MOESM3_ESM.pdf]

nature portfolio

Corresponding author(s): Johannes Algermissen

Last updated by author(s): Dec 9, 2023

Reporting Summary

Nature Portfolio wishes to improve the reproducibility of the work that we publish. This form provides structure for consistency and transparency in reporting. For further information on Nature Portfolio policies, see our Editorial Policies and the Editorial Policy Checklist.

Statistics

For all statistical analyses, confirm that the following items are present in the figure legend, table legend, main text, or Methods section.

n/a	Confirmed	
☐	☒	The exact sample size (*n*) for each experimental group/condition, given as a discrete number and unit of measurement
☐	☒	A statement on whether measurements were taken from distinct samples or whether the same sample was measured repeatedly
☐	☒	The statistical test(s) used AND whether they are one- or two-sided<br>*Only common tests should be described solely by name; describe more complex techniques in the Methods section.*
☐	☒	A description of all covariates tested
☐	☒	A description of any assumptions or corrections, such as tests of normality and adjustment for multiple comparisons
☐	☒	A full description of the statistical parameters including central tendency (e.g. means) or other basic estimates (e.g. regression coefficient) AND variation (e.g. standard deviation) or associated estimates of uncertainty (e.g. confidence intervals)
☐	☒	For null hypothesis testing, the test statistic (e.g. *F*, *t*, *r*) with confidence intervals, effect sizes, degrees of freedom and *P* value noted<br>*Give P values as exact values whenever suitable.*
☐	☒	For Bayesian analysis, information on the choice of priors and Markov chain Monte Carlo settings
☐	☒	For hierarchical and complex designs, identification of the appropriate level for tests and full reporting of outcomes
☐	☒	Estimates of effect sizes (e.g. Cohen's *d*, Pearson's *r*), indicating how they were calculated

Our web collection on statistics for biologists contains articles on many of the points above.

Software and code

Policy information about availability of computer code

Data collection	The experiment was administered using the Psychtoolbox version 3.0.13 in MATLAB R2014b (MathWorks, Natick, MA, United States).
Data analysis	Behavioral data (responses, RTs) were analyzed with R 3.3.2/ Rstudio 1.4.1717 (https://www.rstudio.com/), mostly relying on the packages lme4 version 1.1.26 and afex 0.28.1. (for computing p-values based on likelihood ratio tests). All custom files are shared.<br>Computational modeling was performed with the CBM toolbox (https://github.com/payampiray/cbm, downloaded 2020-01-02) in MATLAB 2018b (MathWorks, Natick, MA, United States). All custom files are shared.<br>fMRI data were analyzed with FSL 6.0.0 (https://fsl.fmrib.ox.ac.uk/fsl/fslwiki). All custom files are shared.<br>EEG data were analyzed with the Fieldtrip toolbox (https://www.fieldtriptoolbox.org/, last time downloaded 2021-11-15) and custom code in MATLAB 2018b (MathWorks, Natick, MA, United States). All custom files are shared.<br>Combined fMRI-informed EEG analyses were performed with the TAfT toolbox (https://github.com/tuhauser/TAfT; heavily adapted) and custom code in MATLAB 2018b (MathWorks, Natick, MA, United States), adapted code is fully shared.<br><br>All code required to achieve the reported results is available under: https://doi.org/10.34973/peg8-xy67. Code will be maintained under https://github.com/johalgermissen/Algermissen2024NatComms, with a permanent copy at the time of publication under https://github.com/denoudenlab/Algermissen2024NatComms.

For manuscripts utilizing custom algorithms or software that are central to the research but not yet described in published literature, software must be made available to editors and reviewers. We strongly encourage code deposition in a community repository (e.g. GitHub). See the Nature Portfolio guidelines for submitting code & software for further information.

Data

Policy information about availability of data

All manuscripts must include a data availability statement. This statement should provide the following information, where applicable:

- Accession codes, unique identifiers, or web links for publicly available datasets
- A description of any restrictions on data availability
- For clinical datasets or third party data, please ensure that the statement adheres to our policy

All raw data is available under: https://doi.org/10.34973/pezs-pw62. In line with requirements of the Ethics Committee and the Radboud University security officer, potentially identifying data (such as imaging data) can only be shared to identifiable researchers. Hence, researchers requesting access to the data have to register and accept a data user agreement; access will then automatically be granted via a “click-through” procedure (without involvement of authors or data stewards).

Preprocessed data and fMRI results have been deposited on the Radboud Repository and are available under: https://doi.org/10.34973/peg8-xy67.
Group-level unthresholded fMRI z-maps are available on Neurovault (https://neurovault.org/collections/11184/).

Research involving human participants, their data, or biological material

Policy information about studies with human participants or human data. See also policy information about sex, gender (identity/presentation), and sexual orientation and race, ethnicity and racism.

Reporting on sex and gender	Gender was self-reported by participants. Gender was only used in the description of the sample (see below: population characteristics), not in any analyses.<br>Given previous literature from animals and human participant samples from other countries, "Pavlovian" or "motivational" biases might constitute a (nearly) "universal" phenomenon shared across different animal species. We did not consider gender effects systematically in our analyses. Doing so would likely require a larger sample size.
Reporting on race, ethnicity, or other socially relevant groupings	No variables on race, ethnicity, and other socially relevant groupings were recorded.<br>Given previous literature from animals and human participant samples from other countries, "Pavlovian" or "motivational" biases might constitute a (nearly) "universal" phenomenon shared across different animal species and different subpopulations of humans.
Population characteristics	The research sample comprised N = 36 healthy young volunteers registered in the research participation system of Radboud University (Mage = 23.6, SDage = 3.4, range 19–32; 25 women; all right-handed; all normal or corrected-to-normal vision). Apart from the inclusion criteria, no efforts were made to recruit specific individuals; this is thus a convenience sample.
Recruitment	We recruited young, healthy human participants via our university-run SONA research participation system (https://radboud.sona-systems.com/). Exclusion criteria comprised claustrophobia, allergy to gels used for EEG electrode application, hearing aids, impaired vision, colorblindness, history of neurological or psychiatric diseases (including heavy concussions and brain surgery), epilepsy and metal parts in the body, or heart problems.<br>Given previous literature from animals and human participant samples from other countries, "Pavlovian" or "motivational" biases might constitute a (nearly) "universal" phenomenon shared across different animal species. We thus believe that the results in this study could have obtained from any human participant. We are not aware of any published evidence hinting at these biases only being present in a subset of participants and/or the neural processes underlying those biases systematically differing across participant groups. Thus, we believe that any form of self-selection bias by our participant group will not have affected the results.
Ethics oversight	CMO2014/288; Commissie Mensengeboden Onderzoek Arnhem-Nijmegen, the Netherlands

Note that full information on the approval of the study protocol must also be provided in the manuscript.

Field-specific reporting

Please select the one below that is the best fit for your research. If you are not sure, read the appropriate sections before making your selection.

☐ Life sciences ☒ Behavioural & social sciences ☐ Ecological, evolutionary & environmental sciences

For a reference copy of the document with all sections, see nature.com/documents/nr-reporting-summary-flat.pdf

Behavioural & social sciences study design

All studies must disclose on these points even when the disclosure is negative.

Study description	Data are quantitative experimental data. Participants performed 640 trials of a Motivational Go/NoGo Learning Task while simultaneous EEG and fMRI data were recorded from them.
Research sample	The research sample comprised N = 36 healthy young volunteers registered in the research participation system of Radboud University (Mage = 23.6, SDage = 3.4, range 19–32; 25 women; all right-handed; all normal or corrected-to-normal vision). Apart from the inclusion criteria, no efforts were made to recruit specific individuals; this is thus a convenience sample.

The participants are representative of individuals affiliated with a Dutch university (students, former students,...). Note that all analyses focused on behavioral and neural data collected from all participants and there was no attempt to define or compare different subgroups of participants.

Sampling strategy	The study sample comprised N = 36 healthy young volunteers. Sample size was based on a highly similar EEG study using the same paradigm (Swart et al., 2018, PLOS Biology) while accounting for some potential drop-out. The study was only visible to participants who fulfilled all inclusion criteria. Participants could voluntarily sign-up for it via our university-run SONA research participation system (https://radboud.sona-systems.com/).
Data collection	The task was performed in a 3T Siemens Magnetom Prisma fit MRI scanner. Participants wore an EEG cap with 64 channels (BrainCap-MR-3-0 64Ch-Standard; Easycap GmbH; Herrsching, Germany; international 10-20 layout, reference electrode at FCz) plus channels for electrocardiogram, heart rate, and respiration. During scanning, two of the authors (JA, JCS), one research assistant and (occasionallly) an MR lab technician were present. Since the study design did not include between-subjects conditions and all within-subjects conditions were manipulated on a trial-by-trial basis, blinding does not apply.
Timing	Data collection took place from March till October 2017.
Data exclusions	Data was collected from N = 36 participants. All participants contributed to behavioral and computational modeling results. For two participants, fMRI functional-to-standard image registration failed; hence, all fMRI-only results are based on 34 participants (Mage = 23.47, 25 women). Four participants exhibited excessive residual noise in their EEG data (> 33% rejected trials) and were thus excluded from all EEG analyses; hence, all EEG-only analyses are based on 32 participants (Mage = 23.09, 23 women). For combined EEG-fMRI analyses, we excluded the above-mentioned six participants plus one more participant whose regression weights for every regressor were about ten times larger than for other participants, leaving 29 participants (Mage = 23.00, 22 women). Exclusions were in line with a previous analysis of this data set (Algermissen et al, 2021, Cerebral Cortex). fMRI- and EEG-only results held when analyzing only those 29 participants (see S01).
Non-participation	No participant dropped out/ declined participation.
Randomization	The study had a within-subject design without any experimental groups; all participants performed all trials/ conditions.

Reporting for specific materials, systems and methods

We require information from authors about some types of materials, experimental systems and methods used in many studies. Here, indicate whether each material, system or method listed is relevant to your study. If you are not sure if a list item applies to your research, read the appropriate section before selecting a response.

Materials & experimental systems

n/a	Involved in the study	
☒	☐	Antibodies
☒	☐	Eukaryotic cell lines
☒	☐	Palaeontology and archaeology
☒	☐	Animals and other organisms
☒	☐	Clinical data
☒	☐	Dual use research of concern
☒	☐	Plants

Methods

n/a	Involved in the study	
☒	☐	ChIP-seq
☒	☐	Flow cytometry
☐	☒	MRI-based neuroimaging

Plants

Seed stocks	*Report on the source of all seed stocks or other plant material used. If applicable, state the seed stock centre and catalogue number. If plant specimens were collected from the field, describe the collection location, date and sampling procedures.*
Novel plant genotypes	*Describe the methods by which all novel plant genotypes were produced. This includes those generated by transgenic approaches, gene editing, chemical/radiation-based mutagenesis and hybridization. For transgenic lines, describe the transformation method, the number of independent lines analyzed and the generation upon which experiments were performed. For gene-edited lines, describe the editor used, the endogenous sequence targeted for editing, the targeting guide RNA sequence (if applicable) and how the editor was applied.*
Authentication	*Describe any authentication procedures for each seed stock used or novel genotype generated. Describe any experiments used to assess the effect of a mutation and, where applicable, how potential secondary effects (e.g. second site T-DNA insertions, mosiacism, off-target gene editing) were examined.*

Magnetic resonance imaging

Experimental design

Design type: task data, event-related design.

Design specifications: Each subject performed 1 session of 640 trials, split in blocks of 110 (blocks 1,2,4,5) or 100 (blocks 3, 6) trials.

Behavioral performance measures: Button presses (yes/no, i.e., Go/NoGo, and exact button pressed) and reaction times (for Go responses) were recorded. Performance above chance was established by assessing the effect of required response on the actual response made.

Acquisition

Imaging type(s): functional

Field strength: 3

Sequence & imaging parameters: whole-brain T2*-weighted sequence, axial-oblique slices, TR = 1400 ms, TE = 32 ms, voxel 614 size 2.0 mm isotropic, interslice gap 0 mm, interleaved multiband slice acquisition with acceleration factor 4, FOV 210 mm, flip angle 75°, A/ P phase encoding direction

Area of acquisition: whole-brain scan

Diffusion MRI: ☐ Used ☒ Not used

Preprocessing

Preprocessing software: All fMRI pre-processing was performed in FSL 6.0.0. After cleaning images from non-brain tissue (brain-extraction with BET), we performed motion correction (MC-FLIRT), spatial smoothing (FWHM 3 mm), and used fieldmaps for B0 unwarping and distortion correction in orbitofrontal areas. We used ICA-AROMA to automatically detect and reject independent components associated with head motion. Finally, images were high-pass filtered at 100 s and pre-whitened.

Normalization: After the first-level GLM analyses, we computed and applied co-registration of EPI images to high-resolution images (linearly with FLIRT using boundary-based registration) and to MNI152 2mm isotropic standard space (non-linearly with FNIRT using 12 DOF and 10 mm warp resolution).

Normalization template: MNI152 2mm isotropic standard space

Noise and artifact removal: We performed ICA-AROMA (Pruim et al., 2015) to detect and regress out independent components related to head motion (non-aggressive denoising). Furthermore, all GLMs contained the 6 motion parameters returned from the registration, the average cerebro-spinal fluid (CSF) and out-of-brain (OOB) signal per volume, and spike regressors for each volume where relative displacement exceeded 2 mm.

Volume censoring: no volume censoring applied

Statistical modeling & inference

Model type and settings: We used 3 mass-univariate GLMs. First-level analyses (block-wise, subject-wise) were fixed-effects, second-level (group-level) analyses mixed-effects.

GLM 1 (model-based GLM): The GLM contains the following 10 regressors: 1-4) 4 regressors crossing the performed action (Go/ NoGo) with the valence of the cue (Win/ Avoid). At the time of cue onset. 5) Response hand: +1 for left and response, 0 for no response, -1 for right hand response. At the time of cue onset. 6) Incorrect response. At the time of responses. 7) Outcome Onset (any outcome). At the time of outcomes. 8) Standard reward prediction errors computed with the standard Q-learning model. 9) Difference between biased and standard reward prediction errors, respectively computed with a) a Q-learning model assuming an increased learning rate for rewarded Go responses and a decreased learning rate after punished NoGo responses, and b) a standard Q-learning model. At the time of outcomes. 10) Invalid outcomes (non-instructed key pressed, returning error message). At the time of outcomes.
In GLMs 1B and 1C, regressors 8 and 9 are computed based on alternative computational models (M8 and M9, respectively).

GLM 2 (model-free GLM): The GLM contains the following 13 regressors: 1-8) 8 regressors crossing the performed action (Go/ NoGo) with the obtained outcome (reward/ no reward = neutral/ no punishment = neutral/ punishment). At the time of outcomes. 9) Left hand response. At the time of responses. 10) Right hand response. At the time of responses. 11) Incorrect response. At the time of responses. 12) Outcome Onset (any outcome). At the time of outcomes. 13) Invalid outcomes (non-instructed key pressed, returning error message). At the time of outcomes.

GLM 3A-C (model-based GLM + EEG regressors): GLM1 regressors 4 performed action x cues valence regressors, response hand, errors, outcome onset, standard prediction errors, the difference term to biased prediction errors, invalid outcomes) plus trial-by-trial EEG (mid-) frontal theta-delta/ alpha/ beta power.

Effect(s) tested: GLM 1: Parametric regressors of standard prediction error and difference to biased prediction error, conjunction of both

Effect(s) tested	contrasts.<br><br>GLM 2:<br>Valence contrast: positive outcomes (regressors 1, 3, 5, 7) minus negative outcomes (regressors 2, 4, 6, 8)<br>Action at time of response: sum of left hand response (regressor 9) and right hand response (regressor 10)<br>Action at time of response: Go regressors (regressors 1-4) minus NoGo regressors (regressors 5-8).<br><br>GLM 3: Parametric regressor of trial-by-trial EEG power

Specify type of analysis: ☒ Whole brain ☐ ROI-based ☐ Both

Statistic type for inference<br>(See Eklund et al. 2016)	Cluster-wise inference using FSL's mixed effects models tool FLAME with a cluster-forming threshold of $z > 3.1$ and cluster-level error control at $\alpha < .05$ (i.e., two one-sided tests with $\alpha < .025$).
Correction	cluster-wise inference

Models & analysis

n/a	Involved in the study
☒	☐ Functional and/or effective connectivity
☒	☐ Graph analysis
☒	☐ Multivariate modeling or predictive analysis
